# Optimal path test data generation based on hybrid negative selection algorithm and genetic algorithm

**DOI:** 10.1371/journal.pone.0242812

**Published:** 2020-11-30

**Authors:** Shayma Mustafa Mohi-Aldeen, Radziah Mohamad, Safaai Deris

**Affiliations:** 1 College of Computer Sciences and Mathematics, University of Mosul, Mosul, Iraq; 2 School of Computing, Faculty of Engineering, Universiti Teknologi Malaysia, Johor, Malaysia; Universiti Sains Malaysia, MALAYSIA

## Abstract

Path testing is the basic approach of white box testing and the main approach to solve it by discovering the particular input data of the searching space to encompass the paths in the software under test. Due to the increasing software complexity, exhaustive testing is impossible and computationally not feasible. The ultimate challenge is to generate suitable test data that maximize the coverage; many approaches have been developed by researchers to accomplish path coverage. The paper suggested a hybrid method (NSA-GA) based on Negative Selection Algorithm (NSA) and Genetic Algorithm (GA) to generate an optimal test data avoiding replication to cover all possible paths. The proposed method modifies the generation of detectors in the generation phase of NSA using GA, as well as, develops a fitness function based on the paths’ prioritization. Different benchmark programs with different data types have been used. The results show that the hybrid method improved the coverage percentage of the programs’ paths, even for complicated paths and its ability to minimize the generated number of test data and enhance the efficiency even with the increased input range of different data types used. This method improves the effectiveness and efficiency of test data generation and maximizes search space area, increasing percentage of path coverage while preventing redundant data.

## Introduction

Software testing is a vital step to improve the quality and increase the reliability of software. The main role of software testing is generating a huge amount of test data which satisfies the sufficiency criteria. Nevertheless, it is a difficult and time-wasting procedure, which accounts for more than half of the software development cost [1]. The whole process can be improved by automation of test data generation, which can significantly reduce time taken for software testing [2, 3]. Due to the nature of the program’s input, the overall testing of the inputs is not feasible for the different program’s size [4]. Among the problems faced by generating test data include non-deterministic or impractical solutions obtained. The highly non-linear structure of the program challenges the search algorithms, making the search for effective and optimum test data of a non-linear, complex, and irregular input in the search space to be difficult [3]. The coverage criteria of structural testing, that has been proposed to decide which of the test data could be cover by the program’s code can be split into two criteria: “control flow coverage” which focuses on testing the program flow, and “data flow coverage” that focuses on the data flow inside the program [5].

There are numerous criteria for control flow coverage like (from weakest to strongest coverage): statement coverage (each statement of the program implemented at least once), branch coverage (where, every branch of the program is achieved at least once with both outcomes), “condition coverage (each condition of the decision in the program is performed)”, “multiple condition coverage (where, all true and false combinations of conditions in compound decisions are exercised at least once)”, and “path coverage (every separate path in the program is exercised at least once)” [1, 2]. Path coverage is the strongest criterion of coverage of the adequacy criteria in the white box testing because each path in a program must be executed at least once [1, 2]. Finding the test data that traverses every path is a complex procedure because there are numerous program’s paths and the process might be endless when the program includes loops [4, 6]. It is also not feasible to find the set of test data which can cover all the nested branches of the program. Accordingly, several researchers proposed various approaches for test data search that can attain maximum path coverage.

Searching of test data in a possible input data set to execute a specific program’s path requires information to guide the search and to determine where the finest test data occur in the searching space. Such a way to deal with the optimization problem [7, 9]. In the past few decades, lots of research has been done to generate test data automatically and several automated test data generation techniques have been suggested in the literature [8–10].

Negative Selection Algorithm (NSA) has been applied to generate a set of test data to cover a programs’ paths [11] but it has some limitations and restrictions in the generation phase that affect its performance. These limitations include random generation of detectors which affects the number of test data generated because the new randomly generated detectors are unable to explore new paths and no benefit can be obtained from existing detectors. Another limitation is the matching rule that calculates the distance between detectors using Hamming distance, and removing all detectors that is close to each other to avoid redundancy. Thus, a hybrid method is proposed to improve the effectiveness of the NSA test data generation method and optimize the generated detectors while increasing the coverage percentage and reducing the overlap between detectors that cover the same paths. This hybrid method is called NSA-GA test data generation method, which is a combination of the NSA with Genetic Algorithm (GA).

The paper is presented as follows. Related works are presented in Section 2, Section 3 and 4 explain the overview of the NSA and GA methods, the proposed algorithm is presented in Section 5, while Section 6 provides details on the experimental evaluations and the conclusion is included in Section 7.

## Related works

Different approaches were used to automatically generate the test data in white-box testing for various criteria of coverage. This is done for increasing the coverage percentage, decreasing the test data number, and to minimize the execution time of the testing process [12, 13]. In general, the techniques of “automatic test data generation” can be categorized into random, symbolic, dynamic, and “search-based test data generation techniques” [1, 4]. In random approach, the selection of test data by chance from an input range. Even though it is easy and can produce many test data rapidly, it also produces an unlimited amount of redundant data [4, 14].

In the early era of software testing automation, the most test data generators use symbolic and dynamic techniques. Symbolic techniques are static, meaning that they specify symbolic as a replacement for real values to variables. Moreover, the dynamic techniques need a real execute of the program by some particular inputs. Whether some of the required test criteria were not fulfilled, then the collected data that obtained through the execution process can be used to choose on which tests best satisfied the set of requirements. This feedback assists the test inputs’ modification gradually till all supplies are fulfilled. Nevertheless, these approaches are impractical, time-wasting, and getting trapped in local optima of the probable input data range in the search space. The troubles also arise when arrays, pointers or loops exist in the “software under test (SUT)” [4, 15]. “Search-based test data generation” is a portion of a broad part of research in the “search-based software engineering” [8, 15]. “Search-based test data generation” involves exploration of the test data in the input range of the software under test in order to fulfill certain test data coverage criterion [9].

Recently, the techniques of “search-based test data generation like Genetic Algorithm (GA), Particle Swarm Optimization (PSO), Ant Colony Optimization (ACO), Simulated Annealing (SA), Artificial Bee Colony (ABC), and Memetic Algorithm (MA)” have been the greatest commonly used techniques to automatically generate the test data, and are used in the meta-heuristic optimizing techniques for finding the top solution for generating the test data by guiding the search to the suitable spaces of the range. Although these evolutionary algorithms could guide the search and produce relevant test data to ensure an ultimate percentage of path coverage, they still need to be enhanced due to their limitations in producing the optimum set of test data [7, 12–14].

The automation of test data generation is a topic that attracts many researchers. Even though numerous methods have been proposed recently to automate the generation of test data to satisfy suitability measures, the implementation of these approaches in finding the optimum solution which satisfies the minimal amount of test data and full cover of the desired measure within an acceptable time while averting redundancy, is quite restricted. In spite of the benefits of these methods, improving the efficiency and effectiveness of the generating method is required, since these methods may produce a large amount of redundant test data, wasting both time and cost. In addition, they may not cover the lower probability execution paths. Problems may also arise from infeasible paths in the program. Mohi-Aldeen et al. (2016) applied negative selection algorithm (NSA) to fulfill the path coverage measure in test data generation. The results are presented that NSA is better than the random approach in reducing the number of test data, preventing duplication and increasing path coverage percentage. But this method its own limitations, namely the random generation of detectors and matching rule which is the distance between the detectors. These limitations affect the effectiveness and efficiency of the test data generation process [11]. To overcome these restrictions, a hybrid method combining NSA with GA is proposed. This technique is able to improve the results in terms of increasing path coverage, reducing the number of test data and decreasing number of generations involved.

### Negative Selection Algorithm (NSA)

**Fig 1:**
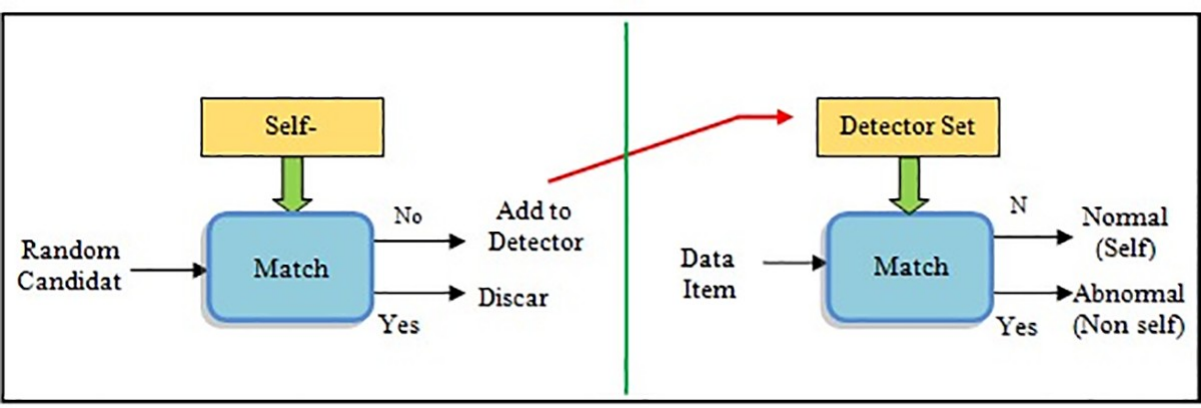
Negative Selection Algorithm (NSA) concepts.

NSA proposed by Forrest is one of the best methods in an Artificial Immune System (AIS) and has been successfully used in wide-ranging applications including “pattern recognition, anomaly detection, computer security, and fault detection” [16, 17]. AIS is a section of a computational intelligence model stimulated by the biologic behavior of “Natural Immune System (NIS)”, a very complicated organic net with a rapid and effective approach of defending the body against a particular external body named antigens [18]. The main concept of NSA is generating a number of detectors in the search space and then uses these detectors in classifying the new data as self or non-self [19]. The algorithm contains two phases, namely “generation stage and detection stage”. In the first stage, a set of “detectors” is produced randomly by some process of censoring that tries to match the self-samples. The corresponding applicants are eliminated, and the rest is kept as “detectors”. This stage is finished after enough detectors (detector’s set) are generated. This termination is determined by certain stopping criteria [19]. Meanwhile, in the stage of detection, the detector set that produced in the first stage is used for checking whether the samples of input corresponding to “self or non-self samples”. If it is matched with any detector, then it is categorized as “non-self”, that means an irregularity in the utmost applications [20]. Fig 1 shows these stages.

### Genetic Algorithm (GA)

**Fig 2:**
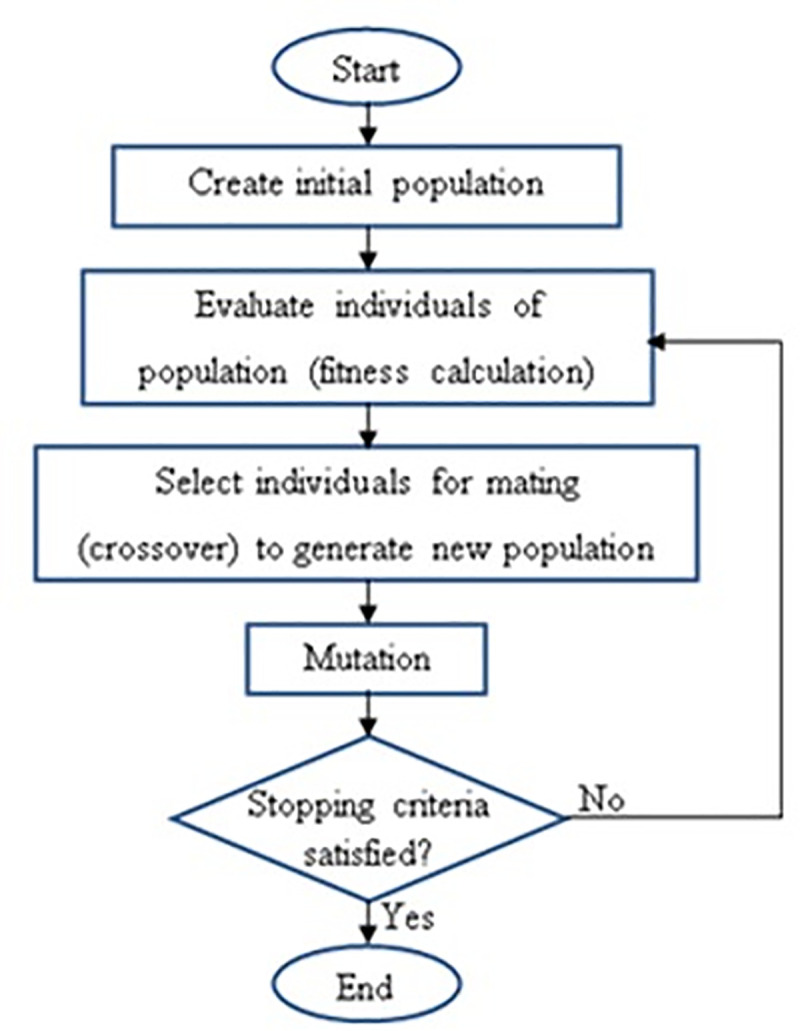
Main idea of Genetic Algorithm (GA) [21].

GA is a computational modeling that simulates the biological development process of the Darwin theory of genetic selection. The basic idea behind GA was introduced by Holland in 1975. GA starts with generating a set of initial individuals which are called chromosome and represented by a binary string generated randomly [21]. GA is a meta-heuristic search method used to solve complex optimization problems which are very difficult to solve using other methods. GA is an ideal solution for optimization problems since it could search very large and highly nonlinear space. GA is computationally simple yet powerful in improving search operations [22]. Three basic operations used in GA are selected, crossover, and mutation. Selection operation is used to select pairs of individuals that will be combined to contribute to the next generation; crossover operation includes two selected chromosomes that are exchanged to produce two offsprings; while the mutation process is used to change one or more proportion of the chromosome. The aim of the mutation process is to preserve the variety in the generation to avoid early convergence into local optimum solution. Since there is no specific way to determine the probability of the mutation, thus it will be determined instinctively. The basic idea of GA is illustrated in (Fig 2).

### The proposed hybrid NSA-GA method

Many factors affect the performance of NSA such as: 1) number of detectors, which affect the efficiency of generation and detection stages and consequently, the speed of the algorithm, 2) detector coverage, that is the section of the non-self space covered by the set of detectors which affect the detection accuracy, and 3) the algorithm, which is used in generating the detectors which affects the quality of the detector set [21, 23]. Several works have been concentrating on the optimization and the limitation of the detectors’ number in generation phase and to improve the matching rule [24, 25]. This paper proposed a hybrid method to modify the generation of detectors in training phase of NSA instead of random generation using GA which can optimize and limit the generations of detectors, because the new randomly generated detectors could not explore a new path and there is no benefit from the existing detectors since the random generation affect the performance, i.e. affecting the coverage space and number of generations needed to reach the coverage criteria. Although the increase in number of detectors improved the coverage, it also increases the number of test data, while the decrease in number of detectors reduced the ability of the method in covering the whole search space, which affects the coverage percentage. Previous work in the NSA test data generation proposed by Mohi-Aldeen et al. (2016) shows that the NSA could not achieve total path coverage for complex programs, and huge amount of test data required for covering the software’s paths. The proposed hybrid NSA-GA method will improve the effectiveness and efficiency of test data generation by providing test data which covers the paths with low execution probability and maximizing the search space area. These moves can increase the percentage of path coverage and prevent data redundancy. NSA detectors generation is a vital role in improving the performance of this algorithm, and the hybrid method modifies the generation of detectors in training phase using GA instead of random generation. This modification can optimize and limit generations of detectors and help in the selection of test data. Another improvement which has been done to NSA is the detector coverage matching rule. Satisfying the detector coverage is critical because the overlap between detectors may affect the percentage of covered paths, and the main goal of fitness function is to improve the detection coverage which will be presented in the next section. The fitness function computes the distance between the candidate detector and all detectors in a detector set. In this paper, a proposed hybridization method is considered to optimize the detectors generated as well as to improve the effectiveness and efficiency of the traditional NSA.

In this method, GA is used to modify the generation of detectors in generation stage instead of randomly generating the detectors. This improvement increases the ability of this hybrid method to optimize and limit the generation of detectors and help in the selection of test data. Moreover, a fitness function based on paths’ prioritization has been used to compel the search to spend more effort on branches that have higher weights (low probability to execute). The fitness function computes the average weight of each path with all detectors in a detector set and maximum fitness function value is used to select the best detector that could cover more space.

#### Generating detectors using GA

Random generation of detectors is the main method that proposed by Forrest, 1994. In this approach, the number of detectors was increased exponentially with the size of the self-set and this increased the time that is needed for completing the process, which is not good and needs to be avoided. Thus, random generation of detectors is not efficient [26, 27]. This result motivates the researchers to use other approaches for generating the detectors set of candidate [21, 28].

**Fig 3:**
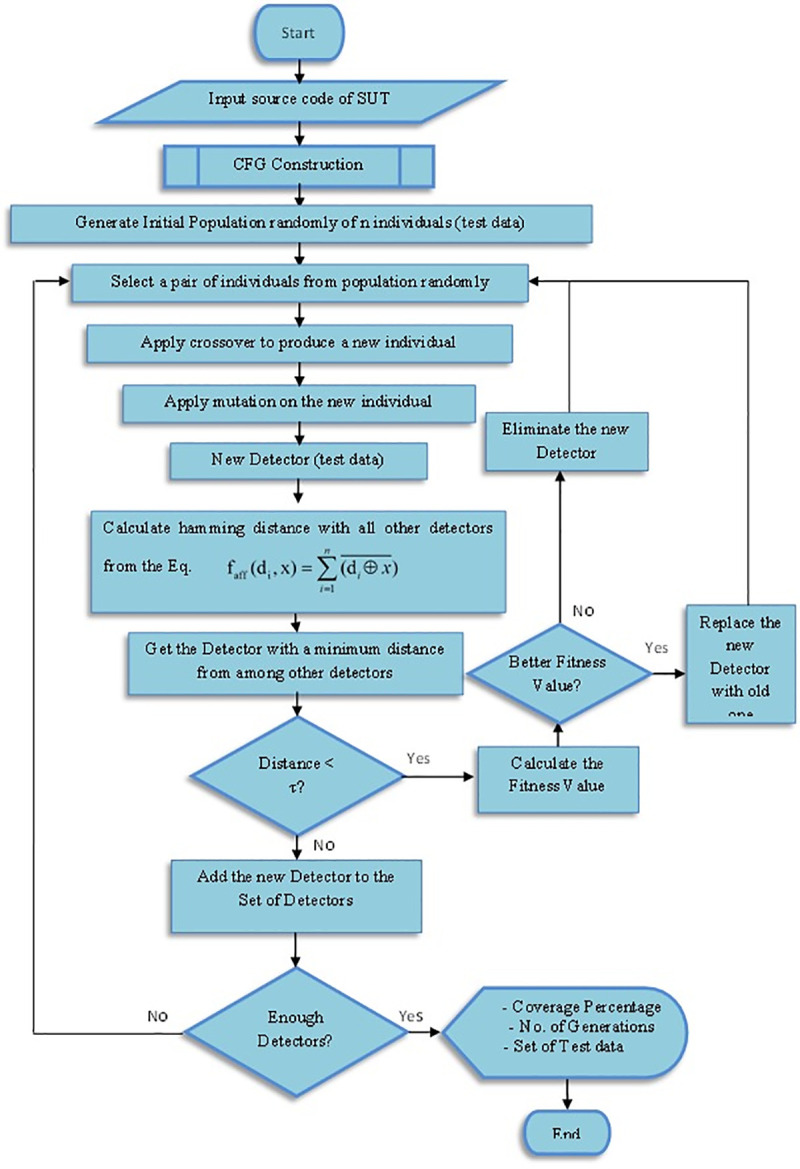
The flowchart of the proposed NSA-GA method.

A modification on the NSA was implemented in this paper by using GA for generating the detectors set that is able to optimize the generated detectors. The chromosome in the proposed method represents the detector, this detector, in turn, represents the values of the program’s input variable represented by a binary string. The chromosome size is equal to the maximum number of the desired detectors and the length of the string depends on the domain length of each input variable. The method begins with the random generation of the initial candidate population of individuals (*x*_1_, *x*_2_,.., *X*_n_) from the search space *S*, where *x* ϵ *S*, and *x* is a test data input which represents the set of input variables. Two parents, *P*_1_ and *P*_2_, are randomly selected and two operators are used which represent the main of the rule of GA. In order to represent the new population, a new individual is created from the selected parents after applying these two operators. During the crossover, the two parents are swapped by applying the single point crossover at a random position of the chromosome to create a new individual (child); this operator happens according to a likelihood of crossover *XP*. Next is the mutation process, which reverse every bit of the new chromosome (changed from 0 to 1 and vice versa) with the pre-defined mutation likelihood *Mp* to develop a new detector *d*_1_. Duplication of the new detector *d*_1_ is checked in the set *D*, if *d*_1_ already exist in the set, remove it from the set *D*; otherwise, compute the distance (Hamming distance) between the new detector *d*_1_ and all detectors *d*_i_ in the set D and the minimum distance obtained will be compared with a threshold value (*τ*). If the distance is less than the threshold value, then this test data is removed, otherwise it is added to the set of test data. This process helps to cover as much of the search area as possible, and here it could cover more paths with limited number of test data for the software under test. Then, select the closest one represented by *d*_2_ and calculate the fitness function of the new detector *d*_1_ and the closest one *d*_2_. If the fitness function of *d*_1_ is greater than *d*_2_, replace the *d*_2_ with *d*_1_, otherwise keep the closest one (replace the closest parent if the fitness is greater). Fig 3 shows the flowchart of the proposed NSA-GA method and the following are the stages of the method:

**Input:**
    *1- The program under test P and its input variable list X = (x*_*1*_, *x*_*2*_, *…*, *x*_*n*_*), where ∀x∈S;*
    *2- The control flow graph (CFG) of P;*
    *3- The constraints of the GA and NSA*, *number of detectors Max*, *τ;*
**Output:**
    *1- Set of test data D = (d*_*1*_, *d*_*2*_, *…*, *d*_*n*_*) which satisfied the path coverage;*
    *2- The set of paths generated U = (u*_*1*_, *u*_*2*_, *…*, *u*_*n*_*);*
**Begin**
  **Step 1:** *Randomly* g*enerate the initial population d*_*1*_ *of n individuals from the search space S;*
  **Step 2:** *While detector number < max or D not reached total coverage of paths U;*
  **Step 3:** *Select a pair of individuals as parents represented by parent 1 and*
      *parent 2;*
  **Step 4:** *Apply crossover to create a new individual represented by child;*
  **Step 5:** *Apply mutation to child;*
  **Step 6:** *Get the child as new detector d*_*1;*_
  **Step 7:** *Remove the redundant test data*:
    *Check the duplication of d*_*1*_ *with d*_*i*_ *in D;*

        Calculate the distance of the new detector d_1_ with every detector d_i_ in D, ∀d_i_∈D by using Hamming distance as below:

$$f_{aff}(d_{i},x)=\sum_{i=1}^{N}(\bar{d_{i}⊕x})$$

  **Step 8:** *Get the detector with minimum distance among other detectors d*_*2*_ *(closest one)*
  **Step 9:** *Cover the maximum search space*:
      *Check the distance f_aff_*(*d*_1_,*d*_2_);
      **If** *f_aff_*(*d*_1_,*d*_2_)*<* *τ* ***then*** *go to step 10;* ***else*,** *add d*_*1*_ *to D*;
  **Step 10:** *Paths prioritization coverage*:
      *Calculate the fitness value of d*_*1*_ *and d*_*2*_ *represented by f*_*1*_ *and f*_*2*_ *respectively;*
  **Step 11:** *Select better detector based on paths prioritization coverage*:
        **If** *(f*_*1*_*> f*_*2*_*) then replace d*_*1*_ *with d*_*2*_ *and return to step 2*;
        ***Else*** *eliminate d*_*2*_ *and return to step 2;*
  **Step 12: End If**
  **Step 13: End If**
  **Step 14: End While**
**End.**

Usually, the probability of crossover is set between 0.6 to 1 while the mutation probability is set to between 0 and 0.1 depending on the experience. These two operator probabilities controlled the population in terms of the exploration and exploitation of the search space [4]. The affinity matching distance (matching threshold) outlines the acceptable difference between detectors and self-samples. The most popular approach used by researchers to choose the most appropriate probability is the trial-and-error approach to select the value that gives the best performance. Then, empirically, the best value of mutation probability which used in the proposed method is 0.01 and 0.8 for crossover probability, while the best value of the threshold used the method was set to 0.5.

#### Paths coverage prioritization

The fitness function evaluates how good a solution is in order to achieve the search goal. The value of an individual fitness represents the measure of its ability to survive in the following generations [4, 29]. The essential role of the fitness function is to compel the search algorithm to spend more effort on the branches that have higher weights based on the priority of the program paths or the most critical paths in the program. Thus, assigning an accurate weight for each branch depending on its reachability is very important. The most important factor in the performance of the NSA is the matching rule which represents the distance between two detectors, and this affects the search space that is covered by detectors set. This fitness function selects the best test data from the set of test data which could cover more paths within the program.

This technique starts with assigning initial weights to all edges of the control flow graph (CFG). The weight value of each edge will decrease by one when a test data that could cover this edge has been generated. The technique will continue to explore new nodes and decrease their weight by one until the end node of the control flow graph is reached. These steps are repeated until there are no more edges in the CFG or there is no more data to traverse the edge. Then the fitness function is calculated from the sum of the total weights of each path from the start node to the end node divided by the length of the path, the result is considered as the objective value of a specific path. Then the path which has the maximum value of fitness function is considered as the most critical or most difficult path and given the highest priority.

The less weight path is the path that traverses with a larger set of test data, so its fitness is decreased, while the path which traverses by less set of test data has the highest weight and higher fitness value. The method will select data which have higher fitness value to be used in the next operation. The fitness function which has been used in our method could adjust the amount of test data dynamically and perform adequate testing for paths with strict inputs. It is also able to produce a particular amount of test data for every path, reduce redundancy of test data and improve the efficiency of generating the test data. The weight of each path is still reduced since there is a possibility of a test data to traverse this path; the more generated test data that traverse the same paths means the least priority of the path, while the path with high weight means higher probability to cover this path. This operation is iterated until all paths are traversed or the maximum number of generations is reached.

## Results and discussion

This section presents the results of the experiments conducted to evaluate the effectiveness of the proposed NSA-GA test data generation method for path coverage. At the beginning, the program is converted automatically into a CFG, and then NSA-GA was used to generate test data automatically. The results show that the proposed method could generate the least amount of test data in less generations’ number and could achieve higher coverage percentage. This result will be likened with both random approach and NSA test data generation method [11] to investigate the performance of the proposed method. The performance is measured in terms of efficiency (represented by the number of generations and amount of test data required to satisfy all paths of the program) and effectiveness (represented by the ratio of paths covered in a program). This section presents the performance of the proposed method for generating test data for the benchmark programs which have been gathered from the previous studies and being commonly utilized as test problems in the recent research of “search-based software testing (SBST)” field.

**Fig 4:**
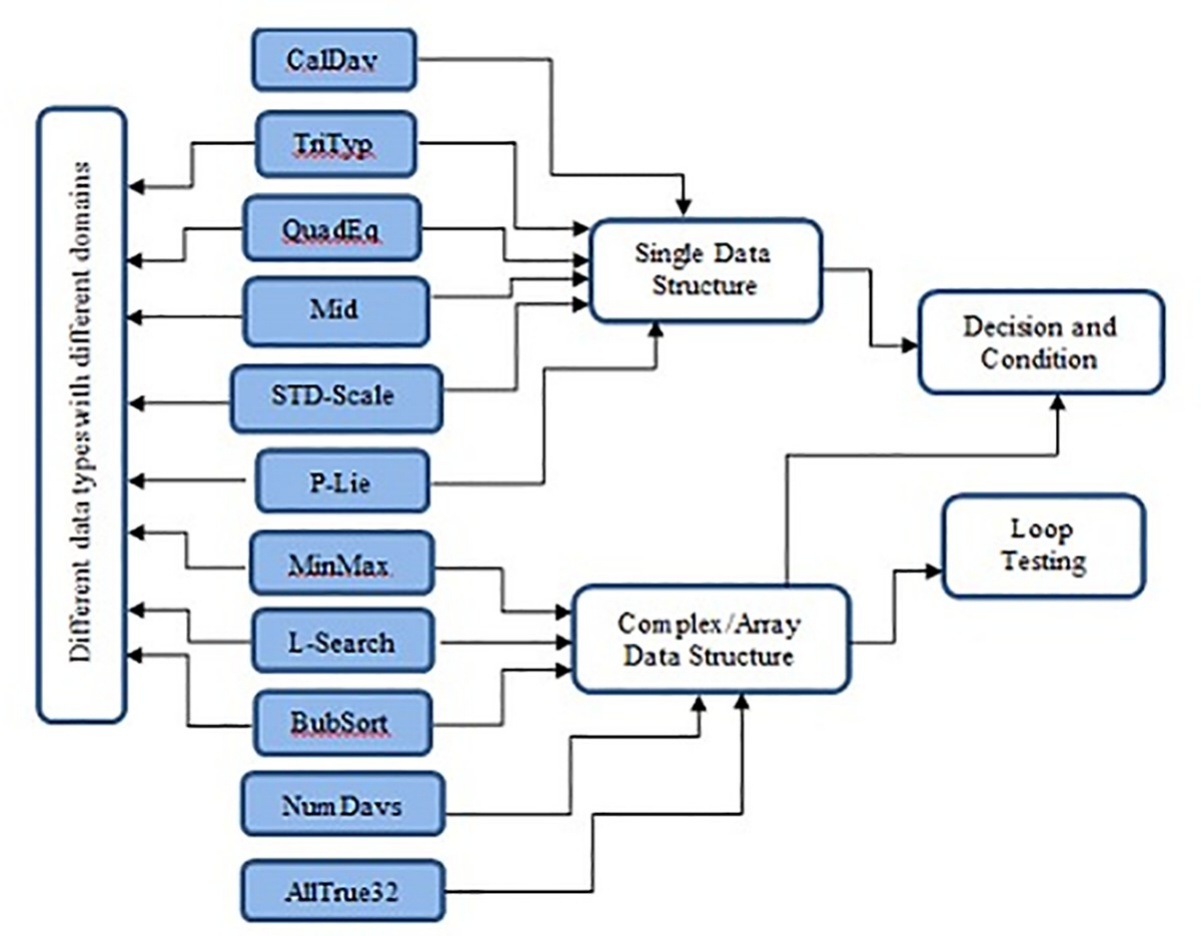
Classification of the benchmarks programs.

The structures of these programs made them suitable to be used to test several test data generation techniques [4, 21]. The programs have different lines of code and complexity (from simple to complex programs) with nested loops, selection, as well as nested selection through the loops. Also, the programs include equality conditions or logical operators, like (=, ! =, <, >, < =, > =) and also complex situations of AND and OR, furthermore the existence of mathematical operators, like (+, -, /, *, Mod), that do the programs suitable for testing numerous test data generation methods. Description of each program is presented in Table 1. The experiments also study the performance of the proposed method with programs which have different types of data, i.e. integers, floats, characters and programs with complex structures, i.e. arrays and loops. The Results of the experiments for different data structures and loops are explained in the next sub heading sections respectively. These programs can be classified based on their program structures as given in (Fig 4). The experiments were executed using Intel® Core™ i7 2.10 GHz, 64 bit processor and 8 GB memory in the Microsoft Windows 7 environment. The implementation of the method is coded using the Delphi 5 platform.

**Table 1:** The benchmark programs description.

Benchmark Shortening	Explanation	Arguments Number	Code Lines	Loops Number	Decision nodes Number	Paths Number	Reference
**TriTyp**	Triangle Type: Find out whether the three input numbers will represent what type of triangle–equilateral, isosceles, scalene, or not a triangle.	3	18	0	6	7	[4]
**Mid**	Find the middle value between three values.	3	20	0	5	6	[6]
**QuadEq**	“Find the quadratic equation root and specify whether it is imaginary or real”.	3	28	0	6	7	[22]
**STD-Scale**	Find the average marks of a student in three subjects.	3	46	0	5	6	[6]
**P-Lie**	Detect “if x and y point locate on x-axis, y-axis or origin”.	2	21	0	3	4	[22]
**MinMax**	Find the maximum and minimum values in an array of integers and real numbers.	1	16	1	2	13	[4]
**L-Search**	“Search a key in array of real, integer, character and string”.	2	27	2	2	20	[4]
**BubSort**	Arrange an array of elements in an increasing order.	1	20	2	1	15	[30]
**NumDays**	Calculate the number of days between two dates.	6	233	4	23	164	[1]
**CalDay**	Determines the “day of the week for a specific date”.	3	119	0	24	25	[31]
**AllTrue32**	Examine if the Boolean elements of an array values are all true.	1	7	1	32	2^32^	[32]

This section illustrates the results of each program. The” triangle type classifier (Trityp)” is considered the utmost well-known program in software testing, which has three input values and is utilized to determine the triangle kind that is characterized as: scalene, isosceles, equilateral or not a triangle. There are twelve branches that contain two AND decision conditions and two OR decisions. The program contains an equality condition and four nesting branches that increase the complication process of checking for the appropriate test data. If every input variable has 2 bytes long, so the space of searching will be huge and its required to execute 2^48^ test cases that equal to 2^16^*2^16^* 2^16^ which is impossible to execute.

**Fig 5:**
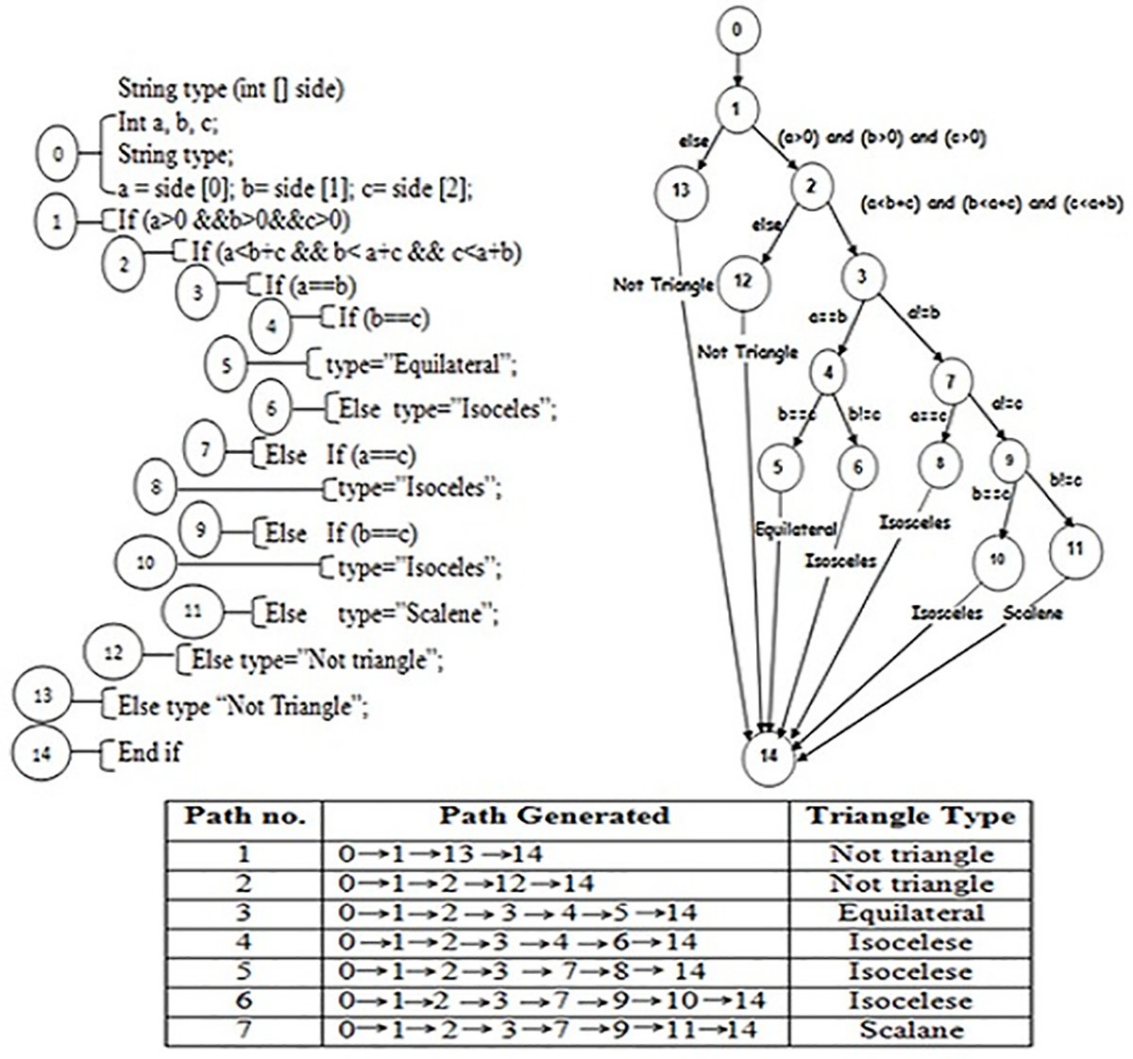
The code and the control flow graph of triangle classification program.

The program appears as an easy program, however, it is not so. A less number of test input data could fulfill the equilateral type of triangle that is the most complicated path to cover because the equilateral triangle path is achieved only and only if the values of the three inputs are positive and equal. “As an example, there are 2^15^–1 types of equilateral triangle. So the chance of randomly choosing three inputs that execute the equilateral path is (2^15^−1 / 2^48^ which is about equal to 1/ 2^33^)”. This made the program a standard for evaluating different approaches in software testing. Fig 5 shows the code and the “control flow graph (CFG)” of this program.

**Fig 6:**
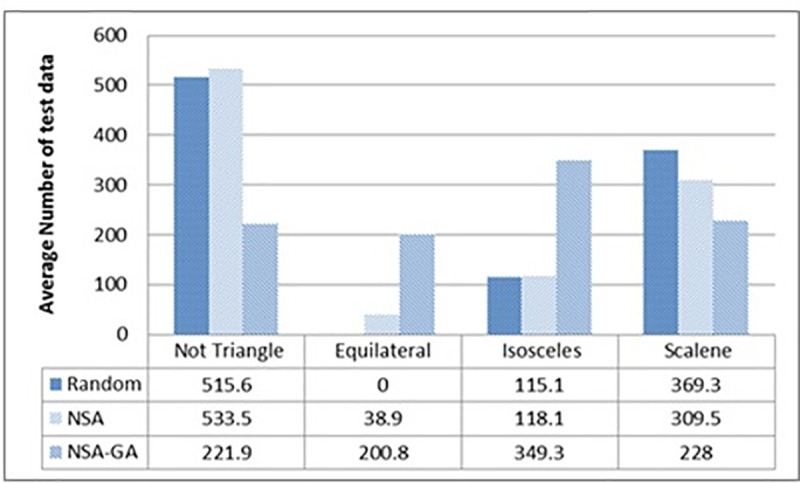
Average amount of test data to fulfill each path of TriTyp program.

For the triangle type classifier (TriTyp), the results show that the NSA-GA is more efficient compared to random testing and NSA. NSA-GA is able to achieve total paths coverage from the second generation while random testing and NSA needed 14 and 3 generations, respectively. The efficiency of the proposed method in generating test data which cover the program paths is presented in (Fig 6). This figure represents the number of test data required to fulfill each path for 1000 iterations. From the figure, compared to random testing or NSA, the proposed NSA-GA method has more probability to generate data for the most difficult path which is the equilateral path because of its capability to direct the searching process to the maximum search space, finding test data to include the low probability paths to execute. The proposed NSA-GA increased the probability of executing equilateral path to 200.8 average test data compared to 38.9 with NSA while random testing failed to traverse this path.

**Fig 7:**
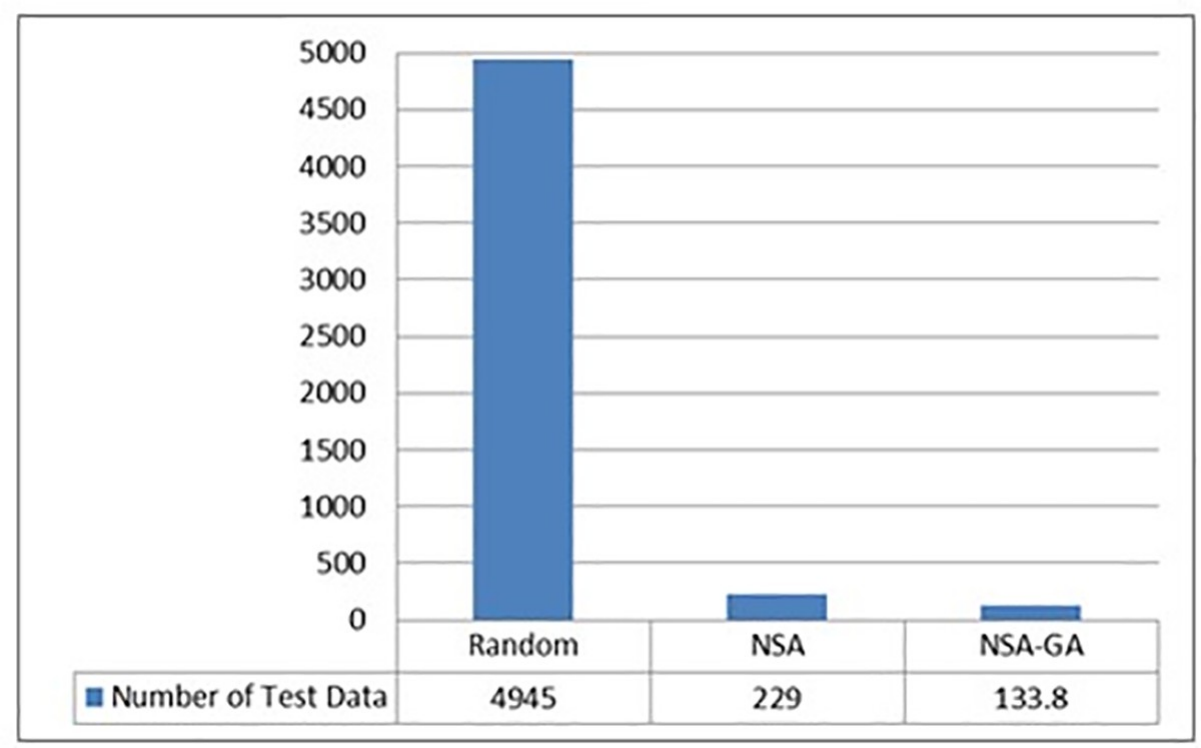
Average amount of test data to fulfill all TriTyp program paths.

Due to the NSA-GA’s ability to remove redundant test data, remove the closest test data and select the test data that reduces intersection, it is able to generate a reduced amount of test data required to fulfill all program paths. Fig 7 shows the ability of the NSA-GA method in reducing the amount of test data needed to fulfill all Trityp program paths. The results show that NSA-GA could fulfill all program paths with minimum amount of test data that is 133.8, while 4945 and 229 test data are needed in random testing and NSA test data generation respectively. Thus, NSA-GA is more efficient than random testing and NSA since it decreases the amount of test data required to fulfill all program paths by more than 95% nd 17% compared to random testing and NSA, respectively.

**Fig 8:**
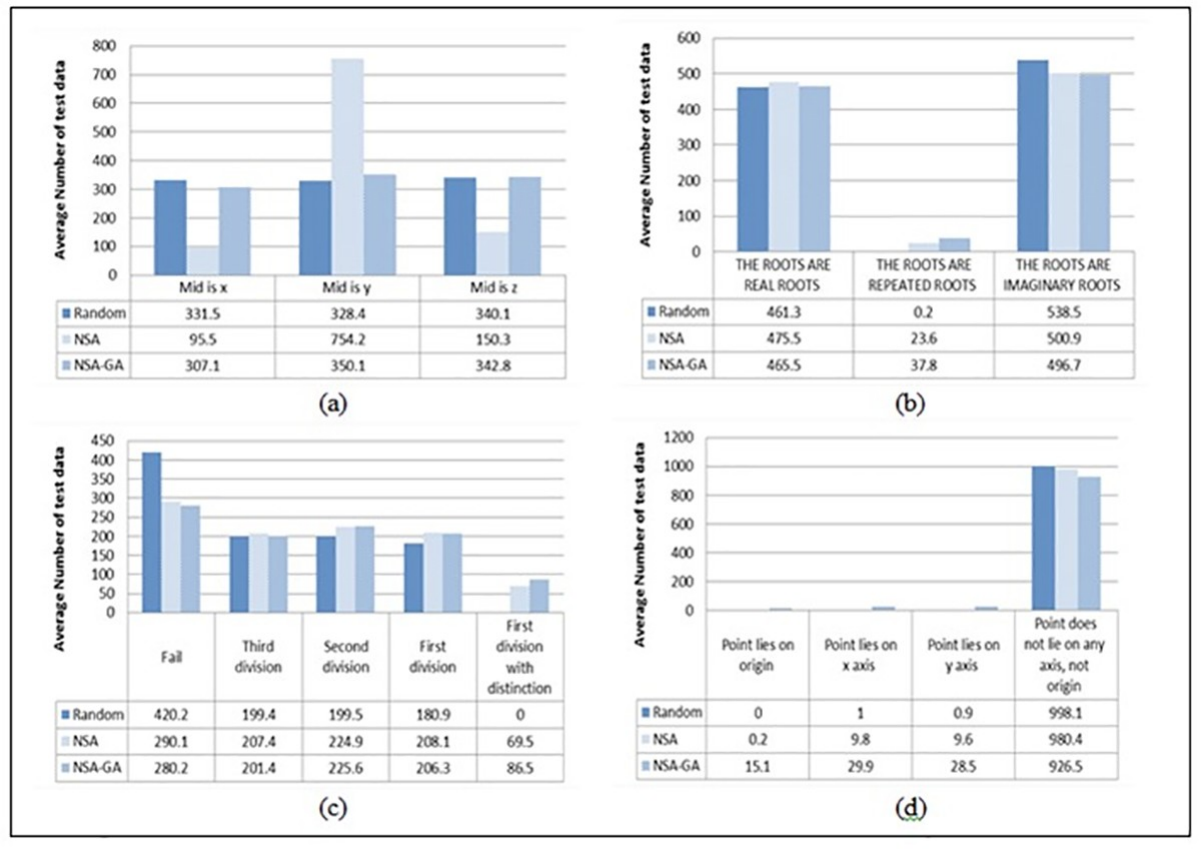
Average amount of test data required to achieve the program paths for ten generations. “(a) Mid program, (b) QuadEq program, (c) STD-Scale program, and (d) P-Lie program”.

Fig 8 presents the average amount of test data required to perform each path of the benchmark programs with 1000 number of iterations using random testing, NSA method, and the proposed NSA-GA. The figures show that the proposed NSA-GA is more efficient than the other two methods in generating the test data for the most difficult paths of the programs since it could maximize the search space that could generate more test data to achieve the low probability execution paths. For example, on average, the amount of test data required to fulfill the most difficult path of the P-Lie program which is the point lies on origin is 15.1 test data for NSA-GA, random testing is unable to cover this path, and NSA recorded 0.2 test data as shown in (Fig 8D).

Another example is the first division with distinction path of the STD-Scale program in (Fig 8C) could not be covered by random testing, NSA needs 69.5 test data to cover this path and NSA-GA has more chance to traverse this path with 86.5 test data. This means that NSA-GA has more ability to generate test data for programs with really difficult paths. The hybrid NSA-GA method is more efficient than random testing and NSA test data generation since it could decrease the amount of test data required to perform all program paths by removing duplicates and closest test data from the set. The results show that the hybrid NSA-GA is more efficient and effective than the other two methods since it has the ability to generate test data to perform all paths of programs with the least number of generations and reduce the amount of test data required.

**Fig 9:**
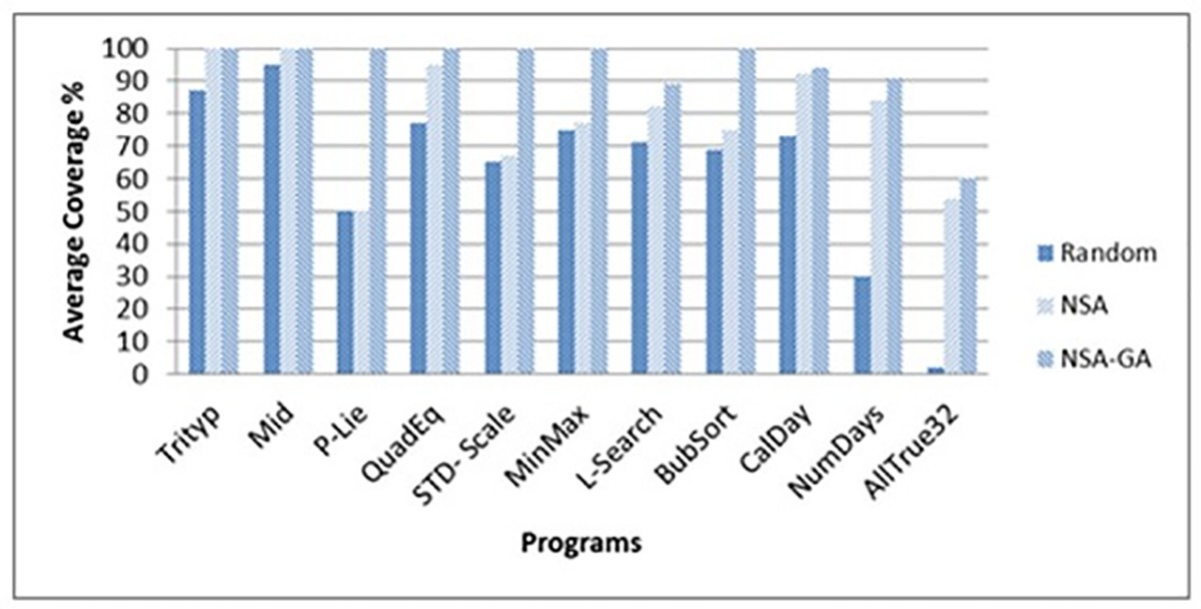
Average coverage percentage for random testing, NSA and NSA-GA methods for all benchmark programs.

**Fig 10:**
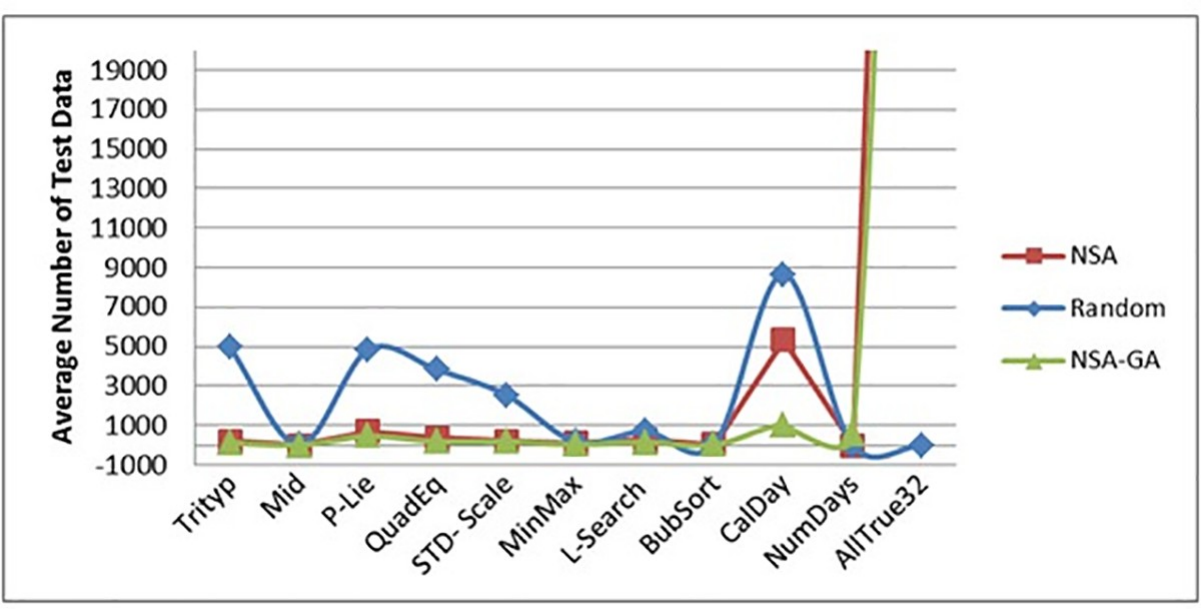
Average number of test data recorded for random testing, NSA and NSA-GA methods for all benchmark programs.

Fig 9 portrays the effectiveness of the methods (in terms of the methods’ coverage percentage) while (Fig 10) shows the efficiency of the methods (in terms of average number of test data needed to cover all paths of the programs). Fig 9 show that the proposed NSA-GA method has highest percentage coverage for most programs. For example, in the P-Lie program, the average coverage percentage (AC) of the proposed NSA-GA is 100% compared to 50% for NSA and random testing. In (Fig 10), NSA-GA employed least number of test data to achieve total coverage. For example, in the CalDay program, the average number of test data required by the NSA-GA to fulfill all paths of this program is 1035.2 with 11 generations, compared to 5338 with 54 generations for NSA and 8658 with 87 generations for random testing.

**Fig 11:**
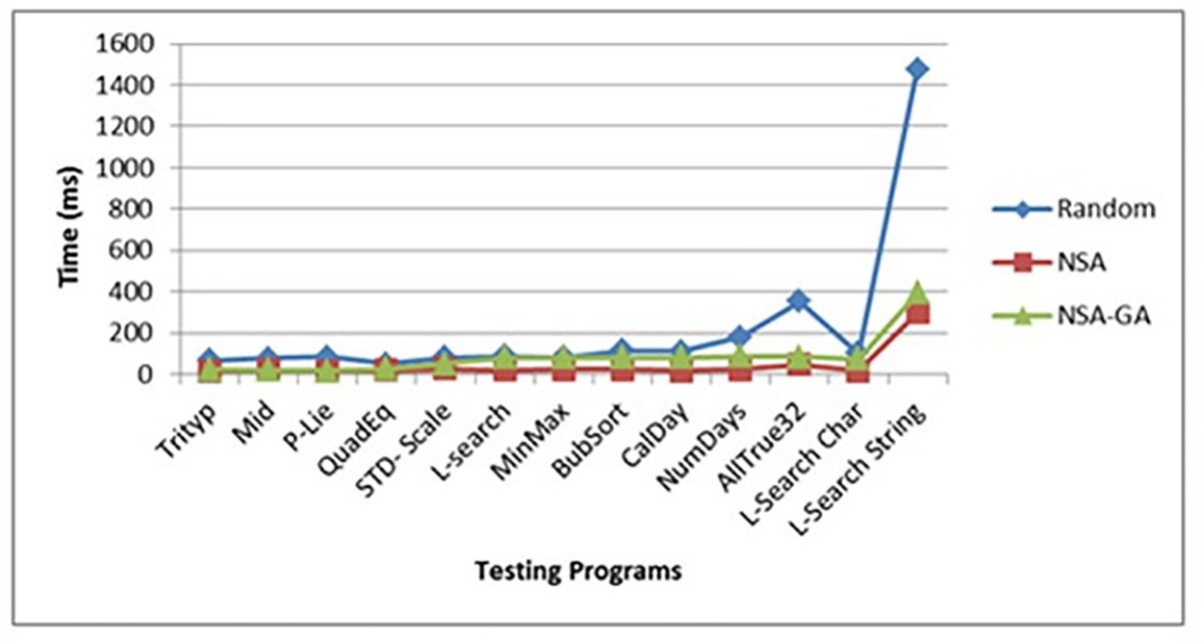
Testing time for random testing, NSA and NSA-GA methods for all benchmark programs.

The average amount of test data needed to perform all P-Lie program paths is 460.1 test data for NSA-GA, while NSA needed 691 test data and random testing needed 4848 test data. This means that the proposed NSA-GA can reduce the amount of test data required to perform all programs paths by more than 34% and 91% compared to NSA and random testing, respectively. Moreover, as portrayed in (Fig 11), NSA-GA is also able to reduce the testing time needed for each program.

### Different data types experiments

This section will present the experimental results of single and complicated data structure (array) with multiple data types, like integer, float, character, and string. The experiments were done on MinMax program that consists array of integers and array of floats, L-Search program that consists array of integers, array of characters, array of floats and array of string, and BubSort program that consists array of integers and array of floats. These programs were used to investigate the performance of the in handling complex data structure and different data types.

The ranges of the input test data in this paper are selected from previous work as (0–100), (0–200), and (0–500) [29]. The results show that the increase in test input data range will gradually decrease the path coverage percentage, increase the number of test data, and generation number required to fulfill all program paths. CalDay and NumDays programs are not considered in this study since they have limited data input representing month and year. Meanwhile, experiment on STD-Scale program was only done on the range (0–100) only because that is the only range applicable to this program. To assess the effectiveness and the efficiency of the suggested approach, the results of NSA-GA method are compared with random approach and NSA test data generation obtained from Mohi-Aldeen et al. (2016) Three performance metrics were considered which are: average amount of test data needed to fulfill all program paths (ATD), average number of generations that required for the whole coverage (AG) and average percentage coverage (AC). The results are presented in Tables 2 and 3. These tables provide a comparison of results for all three methods using integer and float data types with different ranges. For each table, the program name and its range are given in the first and second column; the next three columns contain the AC, ATD, and AG for random testing; the next three columns contain the same measurements using NSA, and the last three columns are for NSA-GA.

**Table 2:** Comparison of results between NSA-GA, NSA, and random testing on all programs using integer data type and different range.

Programs	Data Range	Random			NSA			NSA-GA		
		AC%	ATD	AG	AC%	ATD	AG	AC%	ATD	AG
**TriTyp**	0–100	87	4945	50	100	229	3	100	133.8	2
	0–200	86	19909.4	200	100	554.5	6	100	250.8	3
	0–500	77	53987.3	540	100	589.9	6	100	369.8	4
**Mid**	0–100	95	23	1	100	6	1	100	5	1
	0–200	85	35	1	100	11.5	1	100	8.3	1
	0–500	85	40	1	100	14.1	1	100	7.5	1
**P-Lie**	0–100	50	4848	49	50	691	7	100	460.1	5
	0–200	38	34467.8	345	48	996	10	75	544.1	6
	0–500	28	36104.4	362	35	1063.3	11	63	552	6
**QuadEq**	0–100	78	3816	39	95	362	4	100	85	1
	0–200	75	11252.1	113	93	772.8	8	98	354.7	4
	0–500	70	50955.2	510	93	856.4	9	95	487.2	5
**STD-Scale**	0–100	65	2534	26	67	215	3	100	98	1
**L-Search**	0–100	73	698.6	7	83	290.5	3	89	151.7	2
	0–200	72	721.1	8	82	569	6	84	287.8	3
	0–500	71	1400.4	15	77	577.2	6	82	326	3
**MinMax**	0–100	75	497.2	5	77	113.6	2	100	34.6	1
	0–200	70	633.4	7	77	114.3	2	80	35.9	1
	0–500	70	634.9	7	76	125.7	2	75	36.9	1
**BubSort**	0–100	69	387	4	75	176.8	2	100	14.2	1
	0–200	69	465	5	70	189.2	2	72	17.7	1
	0–500	69	687	7	70	235.5	3	72	19.2	1
**CalDay**	----	73	8658	87	92	5338	54	94	1035.2	11
**NumDays**	----	30	NA	NA	84	NA	NA	91	542.9	6
**AllTrue32**	----	2	NA	NA	54	93966	940	60	81295.3	813

**Table 3:** Comparison of results between NSA-GA, NSA, and random testing on all programs using float data type and different range.

Programs	Data Range	Random			NSA			NSA-GA		
		AC%	ATD	AG	AC%	ATD	AG	AC%	ATD	AG
**TriTyp**	0–100	37	NA	NA	100	559.6	6	100	235.4	3
	0–200	33	NA	NA	100	540.8	6	100	282.2	3
	0–500	29	NA	NA	98	662.4	7	100	415.8	5
**Mid**	0–100	92	12	1	100	7.2	1	100	7.3	1
	0–200	88	14.1	1	100	13.1	1	100	8.8	1
	0–500	87	15.7	1	100	14.2	1	100	10.6	1
**P-Lie**	0–100	30	NA	NA	45	824.7	9	65	490.7	5
	0–200	28	NA	NA	31	978.1	10	57	515.4	6
	0–500	25	NA	NA	28	1031.3	11	50	554.7	6
**QuadEq**	0–100	53	NA	NA	100	738.5	8	100	398	4
	0–200	50	NA	NA	97	864.3	9	100	436.1	5
	0–500	50	NA	NA	95	927.5	10	98	496.9	5
**STD-Scale**	0–100	65	NA	NA	67	NA	NA	83	376	4
**L-Search**	0–100	42	NA	NA	43	NA	NA	52	31185.5	312
	0–200	36	NA	NA	41	NA	NA	46	63355.5	634
	0–500	36	NA	NA	41	NA	NA	43	74370.5	744
**MinMax**	0–100	75	587.2	6	77	127.5	2	78	33.6	1
	0–200	73	773.5	8	76	128.7	2	77	36.3	1
	0–500	69	845.2	9	73	216.8	3	76	37.1	1
**BubSort**	0–100	70	478	5	71	187.4	2	75	18.3	1
	0–200	69	645	7	71	336.7	4	73	18.2	1
	0–500	69	672	7	71	356.5	4	72	20.9	1

From the tables, the results show that the hybrid NSA-GA method employs the least test data number because it has both filter and fitness functions to check the duplication of test data and priority of the paths. It also minimizes the generation number required to perform all programs’ paths. In addition, the proposed hybrid NSA-GA method maximized the percentage coverage of the programs paths due to its ability to maximize search space coverage and traverse low probability execution paths.

As illustrated in Tables 2 and 3, for different input data ranges, the proposed method achieved lowest AG and ATD and highest AC. Although the increase in data range will gradually increase the difficulty of the search test data, NSA-GA still performed better in terms of AG, ATD and AC than the other two methods. This demonstrates that the proposed method is more advantageous to be used for large input range data. For example, when integer data type of (0–100) range is used in the P-Lie program, the AC of the program paths is 50% using random testing and NSA compared to 100% using the proposed NSA-GA method for the same type and range. For cases where the range of input data were increased, for the (0–200) and (0–500) integer data ranges, NSA-GA recorded 75% and 63%, and these percentages are still higher than random testing and NSA for the same ranges. NSA recorded 48% and 35% while random testing recorded 38% and 28%. These percentages decreased when float data type is used, for example, for the same P-Lie program but using float input data type, for the (0–100) data range, the AC is 65% instead of 100%, and the ATD also increased gradually with the increase in data ranges, i.e. 460.1, 544.1 and 552 test data were needed to cover the paths for (0–100), (0–200) and (0–500) data ranges, respectively. However, this number is small compared to other methods.

NSA needs 691, 996 and 1063.3 test data when the range of input data was increased, while random testing needs 4848, 34467.8, and 36104.4 test data. The AG also recorded the same pattern. For float data type, both the ATD and AG also increased with the increase in range. The proposed NSA-GA method recorded the smallest AG compared to random testing and NSA test data generation. For example, for the CalDay program, the AG for NSA-GA is 11 compared to 87 and 54 for random testing and NSA. Meanwhile, for the NumDays program, the full coverage is not achieved (NA) when using random and NSA, but for the NSA-GA it needs six generations to achieve total coverage.

**Fig 12:**
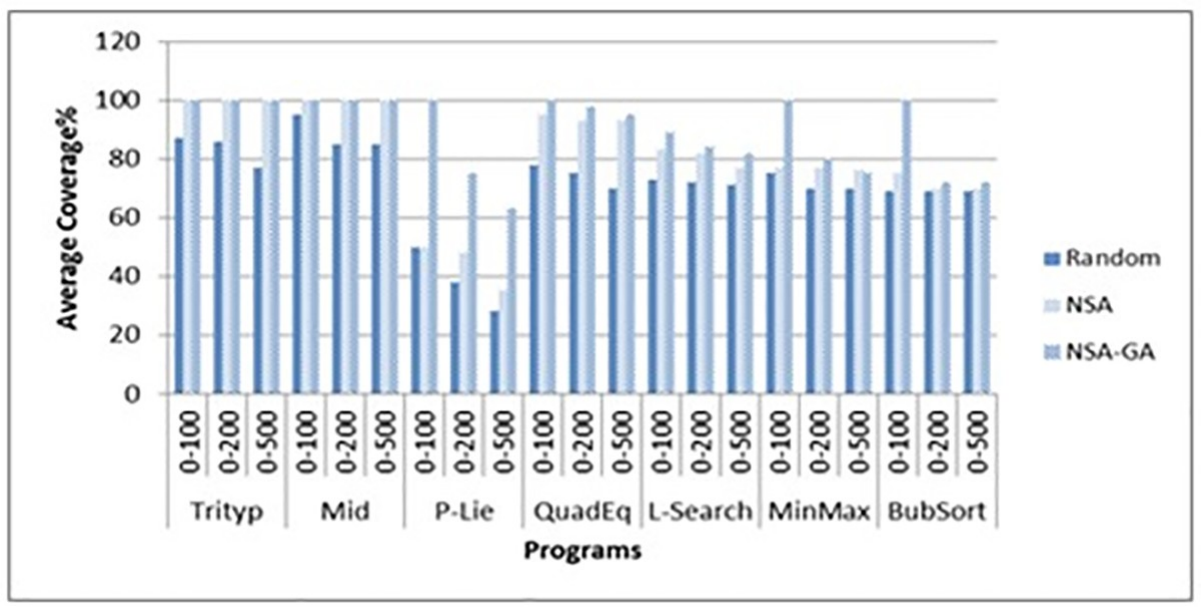
Comparison between AC of random testing, NSA and NSA-GA methods using different ranges for integer input data.

**Fig 13:**
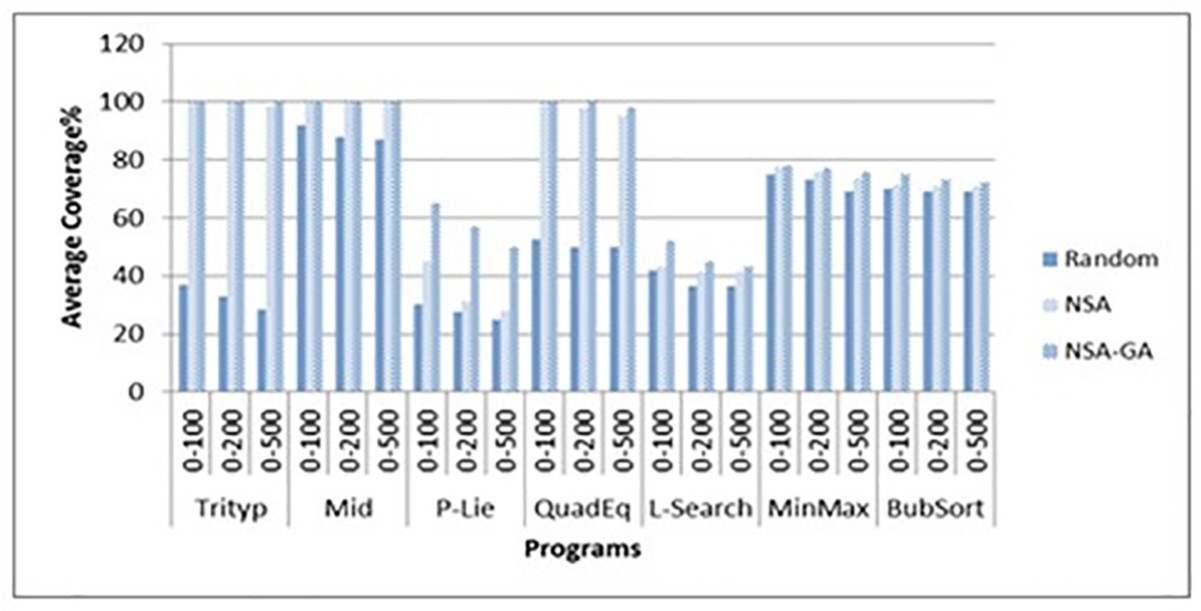
Comparison between AC of random testing, NSA and NSA-GA methods using different ranges for float input data.

The ATD of the proposed method NSA-GA is less than random testing and NSA test data generation. For example, for the MinMax program, the ATD using the proposed method is 34.6 compared to 497.2 and 113.6 for random testing and NSA methods, respectively. Meanwhile, for the case of AC, AC for the proposed NSA-GA is higher than random testing and NSA test data generation. For example, for the STD-Scale program, NSA-GA’s AC is 100 compared to 65 and 67 using random testing and NSA, respectively. After the experiment done, it is found that different data types and different ranges do not affect the proposed NSA-GA method performance. Thus, we could confirm that the proposed NSA-GA is capable of generating best test data for path coverage even using different data types and different ranges. Figs 12 and 13 present AC of each program for integer and float input data types. Figs 12 and 13 show the results of the experiments using the proposed NSA-GA method for both integers and float data types with different ranges. The results show that the performance of the proposed method with floating data is as good as its performance with the integer data, for different ranges and data types (single or array).

In other words, the proposed NSA-GA outperformed the other methods by recording higher coverage percentage and fewer the generated amount of test data with minimum generation’s number and for both data types and in all ranges. Thus, NSA-GA achieved better coverage even using complex data types, complex structures and increase in the search space. From the results, it can be concluded that NSA-GA is the best method in generating a less number of test data to achieve highest path coverage in less number of generations compared to NSA and random testing for all benchmark programs.

### The experiments of loops benchmark programs

This section explains the effectiveness of the proposed method in handling program with loops. Path testing is able to find more logical errors than the statement or branch coverage since the errors exist in several numbers of iterations within the loops. Each number of loop iterations are considered as a different path. The method applies loop testing by executing the loop, zero, one, two and more than two times. The effectiveness of the method was shown using different types of data. Similar to the previous experiments, the results are evaluated based on the average amount of test data required to perform all program paths (ATD), average number of generations that required for the whole coverage (AG) and average percentage coverage (AC). L-Search, MinMax and BubSort programs using different input data types are considered in this section. **In** Table 4, **the programs’ names are given i**n the first column; the next three columns contain the AC, ATD, and AG for random testing; the next three columns contain the same measurements using NSA, and the last three columns are for NSA-GA.

**Table 4:** Comparison between random testing, NSA and NSA-GA methods for programs with loops and different data types.

Programs	Random			NSA			NSA-GA		
	AC%	ATD	AG	AC%	ATD	AG	AC%	ATD	AG
L-Search-Char	90	157.7	2	95	77.5	1	100	52.9	1
L-Search-String	50	625.8	7	78	242.8	3	95	138.5	2
L-Search-Integer	71	698.6	7	82	290.5	3	89	151.6	2
L-Search-Float	41	NA	NA	43	NA	NA	53	31185.5	312
MinMax-Integer	75	497.2	5	77	113.6	2	100	34.6	1
MinMax-Float	69	587.2	6	78	127.5	2	76	33.6	1
BubSort-Integer	69	387	4	74	176.8	2	100	14.2	1
BubSort-Float	69	478	5	71	187.4	2	75	18.3	1
AllTrue32	2	NA	NA	54	93966	940	60	81295.3	813

As can be seen from Table 4, the proposed NSA-GA achieved better results than random testing and NSA test data generation. For AG, NSA-GA achieved total coverage with a minimum number of generations. As an example, the AG of the AllTrue32 program using the proposed method is 813, 940 for NSA, while random testing could not achieve total coverage (NA) for this program. Meanwhile, the AG of the L-Search with floating numbers need 312 generations to fulfill all program paths while neither random testing nor NSA could achieve total coverage NA. For the ATD, ATD of the proposed NSA-GA method is the lowest compared to random testing and NSA. For example, the ATD of L-Search with character input data using NSA-GA is 52.9, but random testing and NSA recorded 157.7 and 77.5, respectively.

**Fig 14:**
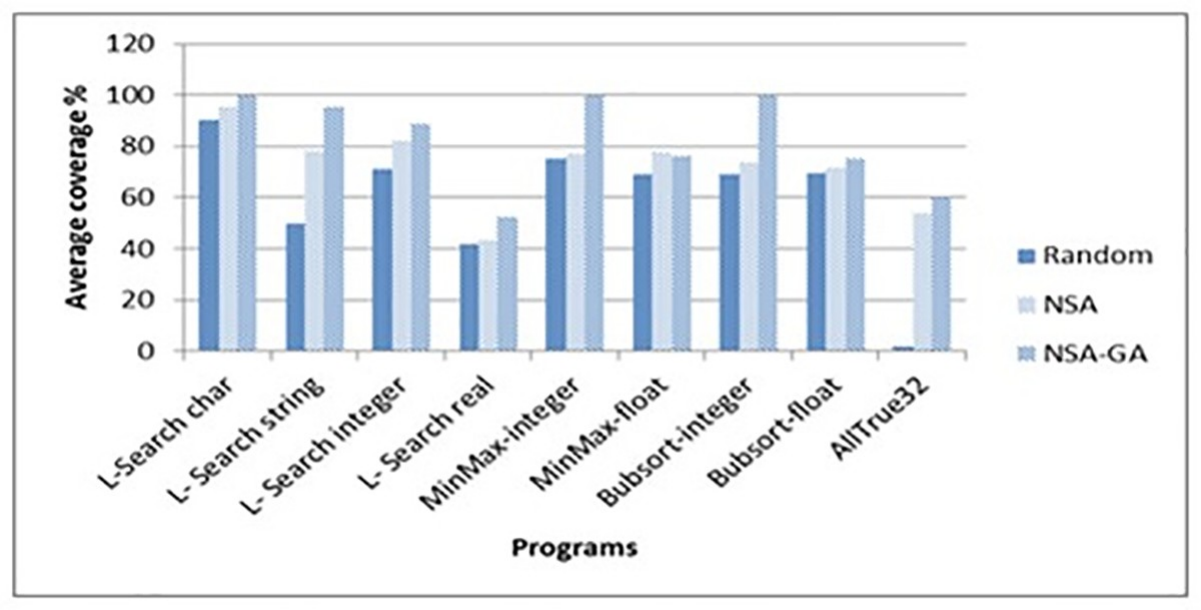
Average coverage of all loops programs with different data types.

In addition, for the AC, the proposed NSA-GA method recorded the highest percentage compared to random testing and NSA test data generation. For example, for the BubSort program, NSA-GA achieved 100% coverage while random testing and NSA only managed to achieve 69% and 74% coverage of total paths. Meanwhile, for AllTrue32 program, the coverage of NSA-GA was 60%, while 2% and 54% were achieved by random testing and NSA. The AC of all programs that contain loops with different input data types has been shown in (Fig 14).

From the results presented in (Fig 14), it can be concluded that the proposed NSA-GA method is suitable to be used in programs that contain complex paths with loops and nested selection since it could achieve total path coverage of the programs as shown in the L-Search-Char, MinMax-Integer and BubSort-Integer compared to NSA and random testing. In addition, it also increases the search coverage.

## Conclusions

The paper proposed a hybrid method NSA-GA which combines NSA with GA. This method modified random generation of detectors in order to generate an optimized and a limited number of detectors (test data set) and direct the search of test data to the paths which have low probability to be executed using fitness functions based on path’s prioritization. The proposed method improves the efficiency and effectiveness of test data generation and maximizes search space area, increasing percentage of path coverage while preventing redundant data.

The results show that the proposed method (NSA-GA) improved the covering of program’s paths, even in the complex paths that contain Nested-If conditions, loops, and multiple types of data with different input ranges. The proposed method produces better results that reduced the amount of generated test data with lesser generations’ number. In other words, NSA-GA’s performance using different data types is as good as its performance with single or array data types. From the results, we can conclude that NSA-GA is the best in generating fewer number of test data that is able to achieve high path coverage in fewer number of generations by comparing with random testing and NSA for all benchmark programs. For the future works, the author suggests to use the statistical validation using ANOVA to validate the results of using the NSA-GA in path testing and also can use another metahurestic algorithm instead of genetic algorithm.

## Supporting information

S1 Dataset (PDF)
