# Supplementary material for: Optimal path test data generation based on hybrid negative selection algorithm and genetic algorithm
Source: PLoS One. 2020 Nov 30;15(11):e0242812. doi: 10.1371/journal.pone.0242812 (PMC7703959; doi:10.1371/journal.pone.0242812)

BENCHMARKS PROGRAMS (DATASET)

The experimental case studies of the research is described which consist of the source code of each test program and followed by its equivalent control flow graph (CFG).

1. Triangle Classifier Source code Program and CFG (`TriTyp`):

1. 0: String type (int [] side)
2. int a, b, c;
3. String type;
4. a = side[0]; b= side[1]; c= side[2];
5. 1: if (a>0&&b>0&&c>0)
6. 2: if (a<b+c&&b<a+c&&c<a+b)
7. 3: if (a==b)
8. 4: if (b==c)
9. 5: type=”Equilateral”;
10. 6: else type=”Isosceles”;
11. 7: else if (a==c)
12. 8: type=”Isosceles”;
13. 9: else if (b==c)
14. 10: type=”Isosceles”;
15. 11: else type=”Scalene”;
16. 12: else type=”Not triangle”;
17. 13: else type=”Not triangle”;
18. 14: end if

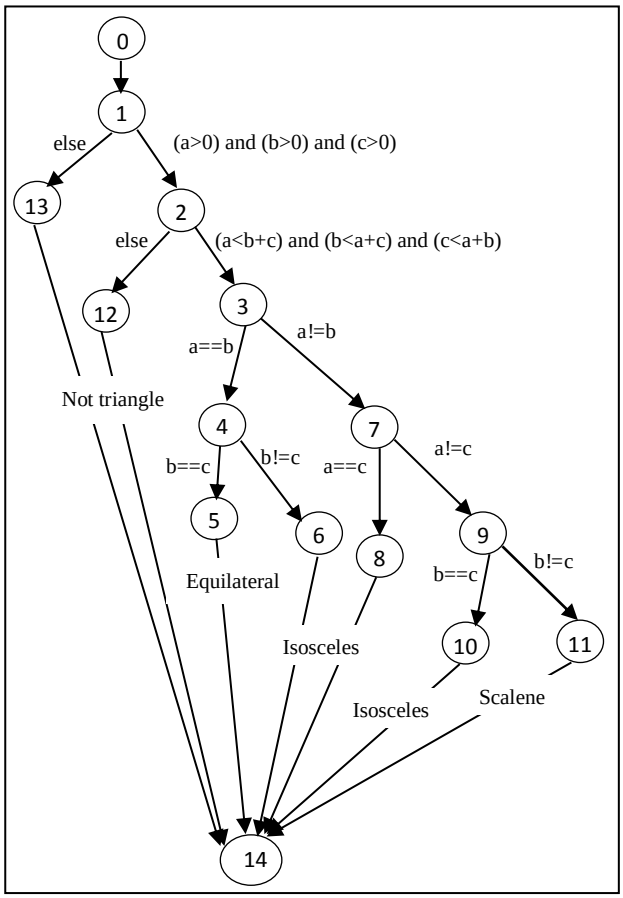

2. Find the Middle Value between Three Values source code Program and CFG (`Mid`):

```
1.  1: int x, y, z;
2.     read x,y,z;
3.      mid= z;
4.  2:  if (y<z)
5.     {
6.  3: if (x<y)
7.  4:  mid=y;
8.  5: else
9.     if (x<z)
10. 6: mid=x;
11. 7:    }
12. 8: Else {
13.     if(x> y)
14. 9:  mid=y;
15. 10: else
16.     if(x>z)
17. 11:  mid=x;
18. 12:    }
19. 13:   Printf(“ mid value=”, mid);
20.       }
```

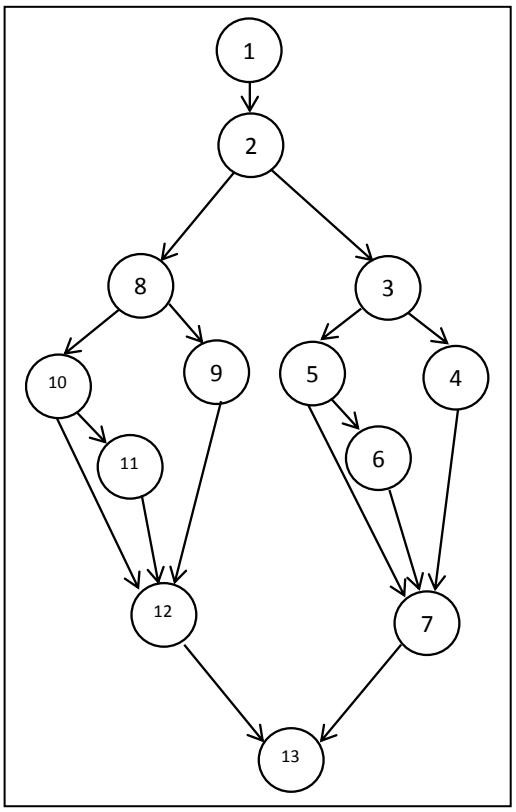

3. Find the Root of Quadratic Equation Source Code Program and CFG (`QuadEq`):

```
1.  1:  int main()
2.      {
3.     int A,B,C;
4.     float disc,deno,x1,x2;
5.     printf ("\n\n PROGRAM TO FIND THE ROOTS OF
6.     A QUADRATIC    EQUATION ");
7.     Printf ("\n\n\t ENTER THE VALUES OF A,B,C");
8.     Scanf ("%d,%d,%d",&A,&B,&C);
9.     disc = (B*B)-(4*A*C);
10.    deno = 2*A;
11. 2: if (disc > 0)
12. 3:  {
13.     Printf ("\n\t THE ROOTS ARE REAL ROOTS");
14.     x1 = (-B/deno)+(sqrt(disc)/deno);
15.     x2 = (-B/deno)-(sqrt(disc)/deno);
16.     printf ("\n\n\t THE ROOTS ARE...: %f and %f\n",x1,x2);
17.     }
18. 4: else
19.     if(disc == 0)
20. 5: {
21.    Printf ("\n\t THE ROOTS ARE REPEATED ROOTS");
22.     x1 = -B/deno;
23.     printf ("\n\n\t THE ROOT IS...: %f\n",x1);
24.      }
25. 6: else
26.    Printf ("\n\t THE ROOTS ARE IMAGINARY ROOTS\n");
27. 7:  printf ("\n-----------------------------------------------------");
28.      Getch ();
29.     }
```

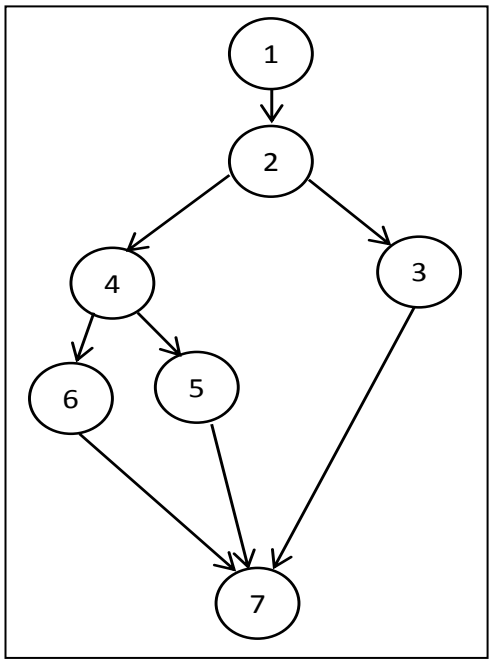

4. Student Scale Source Code Program and CFG (`Std-Scale`):

```
1.  #include<stdio.h>
2.  #include<conio.h>
3.  1: void main()
4.     {
5.      int mark1, mark2,mark3,avg;
6.      clrscr();
7.      printf("Enter marks of 3 subjects (between 0-100)\n");
8.      printf("Enter marks of first subject:");
9.      scanf("%d", &mark1);
10.     printf("Enter marks of second subject:");
11.     scanf("%d", &mark2);
12.     printf("Enter marks of third subject:");
13.     scanf("%d",&mark3);
14. 2: if(mark1>100||mark1<0||mark2>100||mark2<0||mark3>100||mark3<0)
15.      {
16.  3: printf("Invalid Marks! Please try again");
17.      }
18. 4: else
19.     {
20.      avg=(mark1+mark2+mark3)/3;
21. 5: if(avg<40)
22.     {
23. 6: printf("Fail");
24.     }
25. 7: else
26.     if(avg>=40&&avg<50)
27.      {
28. 8: printf("Third Division");
29.      }
30. 9: else
31.       if(avg>=50&&avg<60)
32.        {
33. 10: printf("Second Division");
34.         }
35. 11: else
36.       if(avg>=60&&avg<75)
37.        {
38. 12: printf("First Division");
39.        }
40. 13: else
41.       {
42.         printf("First Division with Distinction");
43.        }
44. 14: }
45. 15: getch();
46.       }
```

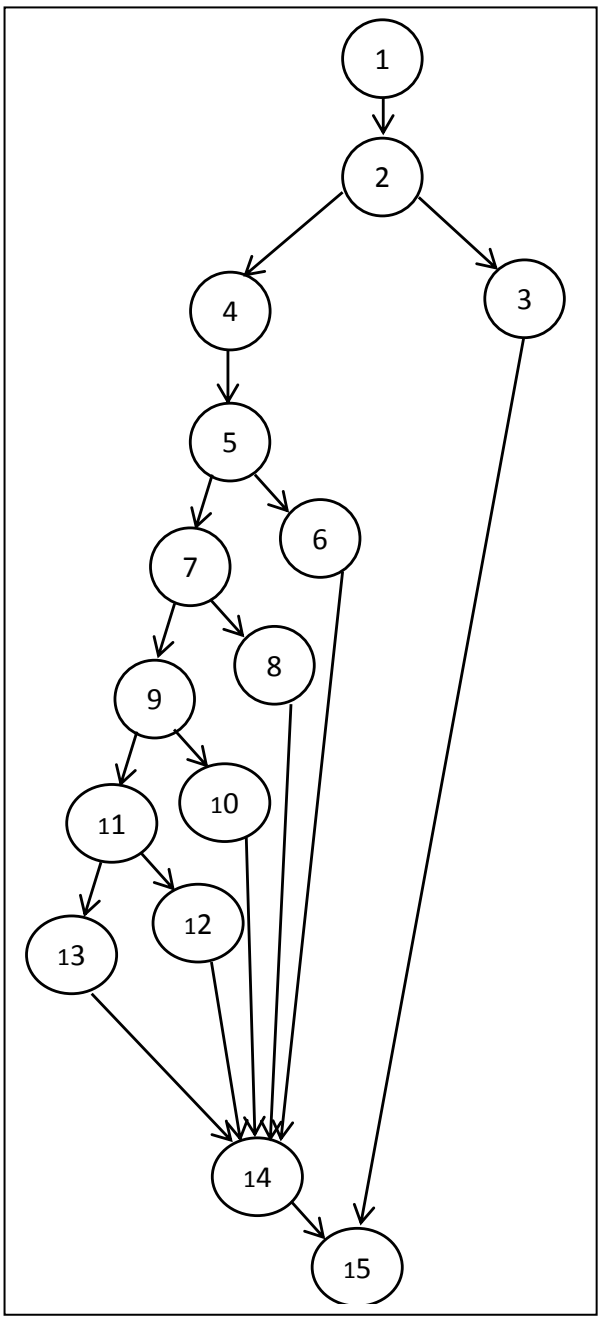

5. Find the Position of a Point if Lie on X- axis, Y-axis or Origin Source Code Program and CFG (`P_Lie`):

```
1: # include<stdio.h>
   # include<conio.h>
   void main()
   {
   int x,y;
   clrscr();
   printf("\nEnter the x and y coordinates of a point:");
   scanf("%d%d",&x,&y);
2: if(x == 0 && y == 0)
3:  printf("Point lies on origin");
4:  else
     if(x == 0 && y!== 0)
5: printf("\nPoint lies on y axis");
6: else
    if(x!=0 && Y== 0)
7: printf("\nPoint lies on X axis")
8: else
    printf("\nPoint dose not lie on any axis, nor origin");
9:  printf("\n press any key to exit.”);
    getch();
     }
```

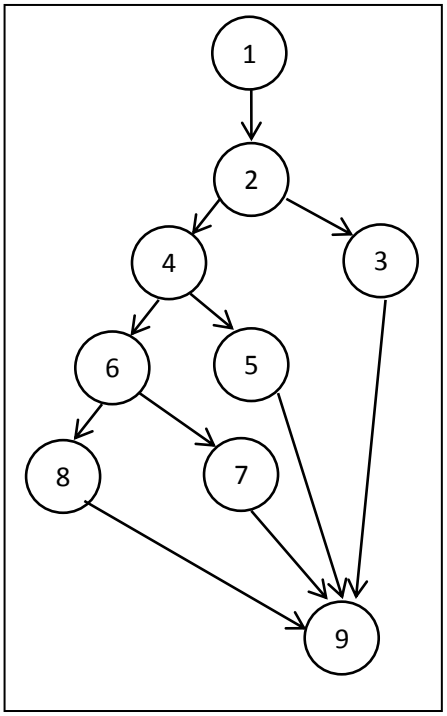

6. Find Minimum and Maximum in array Source Code Program and CFG (`MinMax`):

```
1. 1: function [miniMaxi] = minimaxi(num)
2.     numLength = length(num);
3.     mini = num(1);
4.     maxi = num(1);
5.     idx = 2;
6. 2: while (idx <= numLength) % Branching #1
7. 3: if maxi < num(idx) % Branching #2
8. 4:   maxi = num(idx);
9. 5: end
10. 6: if mini > num(idx) % Branching #3
11. 7: mini = num(idx);
12. 8: end
13. 9: idx = idx+1;
14.    end % while end
15. 10: miniMaxi = [mini maxi];
16.     end
```

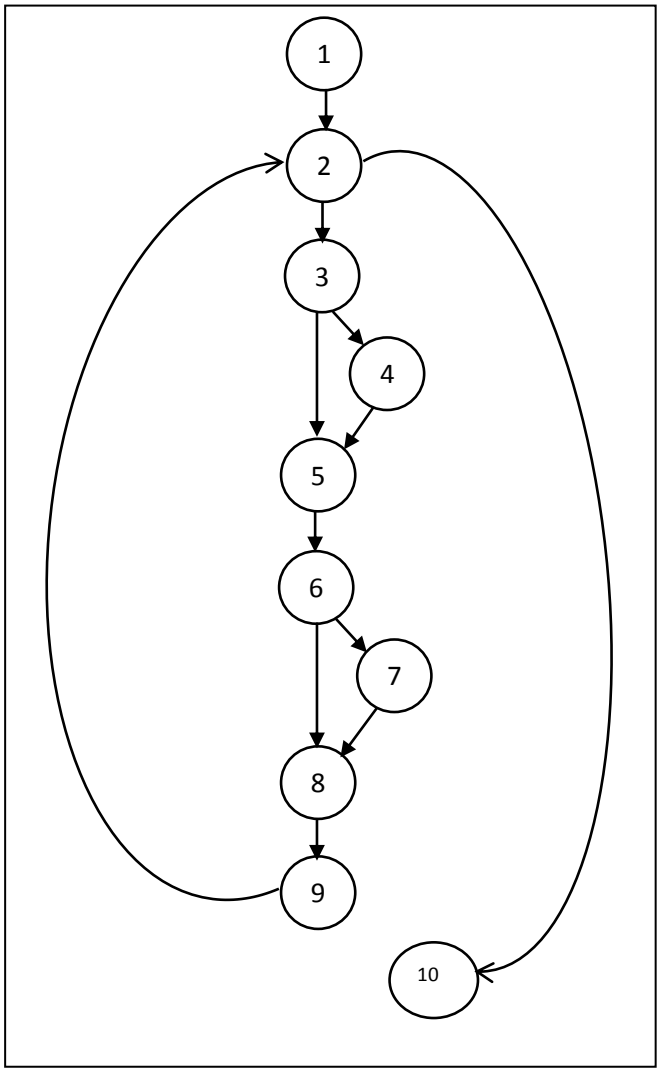

7. Search for a key in an array using Linear Search Source Code Program and CFG (`L-Search`):

```
1.  1: import java.util.Scanner;
2.     class LinearSearch
3.     {
4.     public static void main(String args[])
5.     {
6.     int c, n, search, array[];
7.     Scanner in = new Scanner(System.in);
8.     System.out.println("Enter number of elements");
9.     n = in.nextInt();
10.    array = new int[n];
11.    System.out.println("Enter " + n + " integers");
12. 2: for (c = 0; c < n; c++)
13. 3:   array[c] = in.nextInt();
14. 4: System.out.println("Enter value to find");
15.    search = in.nextInt();
16. 5: for (c = 0; c < n; c++)
17.    {
18. 6: if (array[c] == search) /*Searching element is present*/
19. 7: {
20.    System.out.println(search + " is present at location "
21.     + (c + 1) + ".");
22.    break;
23. 8: }
24.    }
25. 9: if (c == n)  /* Searching element is absent */
26. 10: System.out.println(search + " is not present in array.");
27. 11: }
28.      }
```

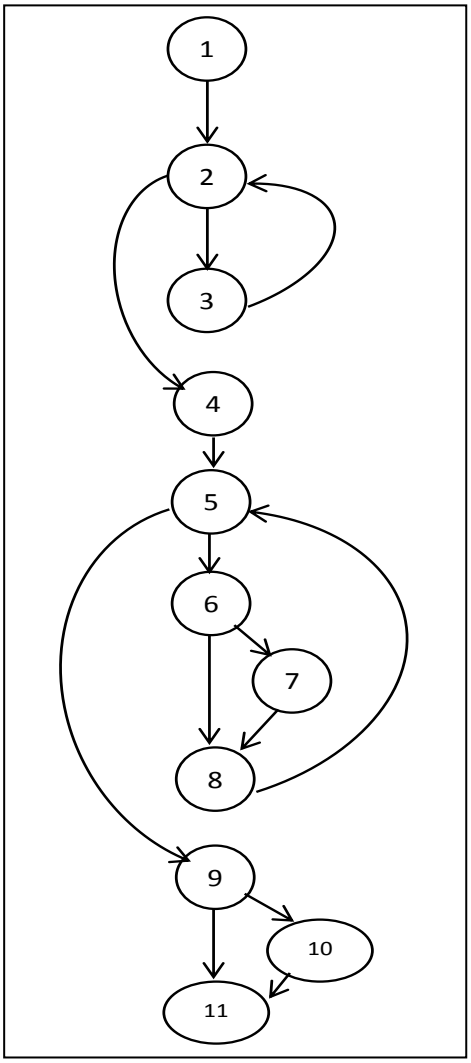

8. Sort an Array of Elements using Bubble Sort Source Code and CFG (`BubSort`)

```
1. 1: function [sortedArray] = bubble(anyArray)
2.     sorted = 0; % 0 means false
3.     i = 1;
4.     n = length(anyArray);
5. 2: while ((i <= (n-1)) && ~sorted) % Branch # 1
6. 3:  sorted = 1;
7.     j = n;
8. 4:  for j=n:-1:i+1 % Branch # 2
9. 5:  if (anyArray(j) < anyArray(j-1)) % Branch # 3
10.    %exchange(anyArray(j), anyArray(j-1));
11. 6: temp = anyArray(j);
12.    anyArray(j) = anyArray(j-1);
13.    anyArray(j-1) = temp;
14.    sorted = 0;
15. 7: end
16. 8: end
17. 9: i = i + 1;
18. 10: end
19. 11: sortedArray = anyArray;
20.     end
```

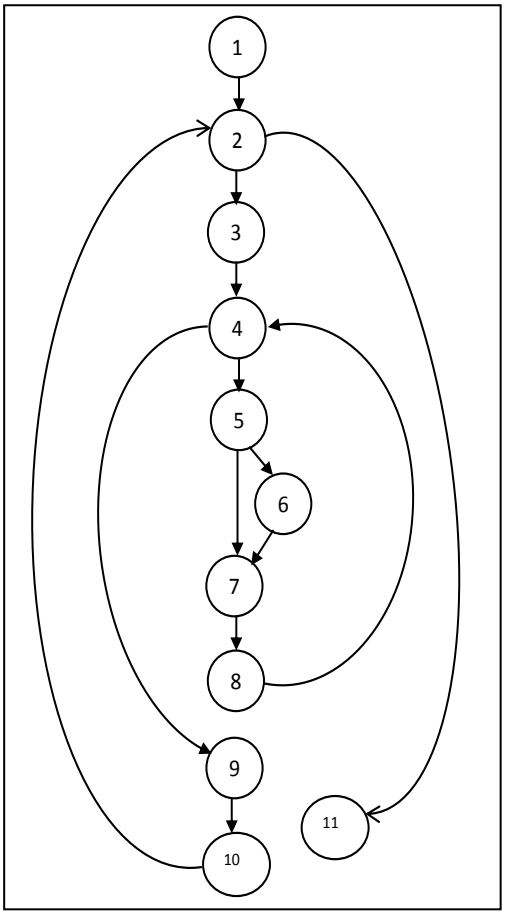

9. Calculating Number of Days between Two Dates Source Code Program and CFG (`NumDays`)

```
001    // Days in-between dates
002    // Compiled in Visual C++ 2008 Express Edition
003       // Language: C++/STL
1: 004    #include<iostream>
005    using namespace std;
006    int main()
007    {
008       int days_in_months[] = {31, 28, 31, 30, 31, 30, 31, 31, 30, 31, 30, 31};
009          int first_day, second_day;
010          int first_month, second_month;
011          int first_year, second_year;
012          int years_difference, days_difference;
013          int months_total;
014          int reg_year = 365;
015       cout<<"Program to calculate how many days are in between the
          day/month/year entered."<<endl;
016          cout<<endl;
017          cout<<"Please enter the date by day, month, year."<<endl;
018          cout<<endl;
019          cout<<"First date:: "<<endl;
020          cout<<endl;
021          cout<<"Day: ;
022          cin>>first_day;
2: 023          if(first_day > 31 || first_day <= 0)
3: 024          {
025             cout<<"Incorrect day entered"<<endl;
026             cin.ignore();
027             return 0;
028          }
4: 029          cout<<"Month: ";
030             cin>>first_month;
5: 031          if(first_month > 12 || first_month <= 0)
6: 032          {
033             cout<<"Incorrect Month entered"<<endl;
034             cin.ignore();
035             return 0;
036          }
```

```
7: 037        cout<<"Year: ;
038           cin>>first_year;
8: 039        if(first_year > 9999 || first_year < 0)
9: 040        {
041           cout<<"Incorrect Year Entered"<<endl;
042           cin.ignore();
043           return 0;
044        }
045
10: 046          cout<<endl;
047        cout<<"\nSecond date:: "<<endl;
048        cout<<endl;
049
050        cout<<"Day: ";
051             cin>>second_day;
11: 052        if(second_day > 31 || second_day <= 0)
12: 053        {
054               cout<<"Incorrect day entered"<<endl;
055           cin.ignore();
056           return 0;
057             }
13: 058        cout<<"Month: ";
059             cin>>second_month;
14: 060        if(second_month > 12 || second_month <= 0)
15: 061        {
062               cout<<"Incorrect Month entered"<<endl;
063           cin.ignore();
064           return 0;
065             }
16: 066        cout<<"Year: ";
067             cin>>second_year;
17: 068        if(second_year > 9999 || second_year < 0)
18: 069        {
070               cout<<"Incorrect Year Entered"<<endl;
071           cin.ignore();
072           return 0;
073        }
074
075
```

```
076     ////////////////////////////Years////////////////////////////////
077
078
19: 079       if(first_year == second_year)
20: 080       {
081         years_difference = 0;
082     }
21: 083       else
084       {
085           if(first_year % 4 == 0 && first_year % 100 != 0 || first_year %
 400 == 0)
22:
086           {
087       if(second_year % 4 == 0 && second_year % 100 != 0 || second_year
 % 400 == 0)
23:
088             {
089                 if(first_year > second_year)
24: 090             {
091               years_difference = (first_year - second_year) * (reg_year) + 2;
092                 }
25: 093             else
094                   {
095               years_difference = (second_year - first_year) * (reg_year) + 2;
096                 }
26: 097             if(second_month > first_month)
27: 098               {
099                 if(days_in_months[first_month - 1] > days_in_months[1])
28: 100             {
101               --years_difference;
102                 }
29: 103             }
30: 104           }
31: 105           else
106               {
107                 if(first_year > second_year)
32: 108           {
109               years_difference = (first_year - second_year) * (reg_year) + 1;
110                 }
33: 111             else
```

```
112                     {
113                    years_difference = (second_year - first_year) * (reg_year) + 1;
114                     }
34: 115                 if(first_month > second_month)
35: 116                   {
                          if(days_in_months[second_month - 1] >
117          days_in_months[1])
36:
                          {
118
119                      --years_difference;
120                       }
37: 121                 }
122                  }
123               }
38: 124           else
125            {
126               if(first_year > second_year)
39: 127            {
128                     years_difference = (first_year - second_year) * (reg_year);
129                  }
40: 130              else
131               {
132                  years_difference = (second_year - first_year) * (reg_year);
133                  }
41: 134           }
 135         }
136
137         ////////////////////////////Months/////////////////////////////////
138
139
42: 140         if(first_month == second_month)
43: 141         {
142               months_total = 0;
143         }
44: 144         else
145         {
146            if(first_month > second_month)
45: 147           {
148                  for(int i = (first_month - 1); i > (second_month - 1); i--)
46: 149              {
```

```
150                     static int months_total_temp = 0;
151                 months_total_temp += days_in_months[i];
152                 months_total = months_total_temp;
47: 153               }
154               }
48: 155           else
156               {
157                 for(int i = (first_month - 1); i < (second_month - 1); i++)
49: 158             {
159                 static int months_total_temp = 0;
160                 months_total_temp += days_in_months[i];
161                     months_total = months_total_temp;
50: 162             }
163               }
51: 164       }
165
166       ////////////////////////Days////////////////////////////////
167
168       int days_total;
169
52: 170         if (first_day == second_day)
53: 171         {
172               days_difference = 0;
173           days_total = (years_difference + months_total) - days_difference;
174       }
54: 175         else
176           {
177               if(first_day > second_day)
55: 178           {
179             days_difference = first_day - second_day;
180             days_total = (years_difference + months_total) - days_difference;
181               }
56: 182           else
183           {
184             days_difference = second_day - first_day;
185             days_total = (years_difference + months_total) + days_difference;
186           }
57: 187         }
188
```

```
189     //////////////////////////In Between Leap Years//////////////////////////////
190
58: 191       if(first_year == second_year)
59: 192       {
193           }
60: 194       else
195     {
196        if(first_year > second_year)
61: 197           {
198              for(int i = (second_year + 1); i < first_year; i++)
62: 199           {
200                 if(i % 4 == 0 && i % 100 != 0 || i % 400 == 0)
63: 201              {
202                    cout<<endl;
203               cout<<i<<endl;
204               ++days_total;
64: 205              }
65: 206           }
207           }
66: 208           else
209        {
210           for(int i = (first_year + 1); i < second_year; i++)
67: 211           {
212              if(i % 4 == 0 && i % 100 != 0 || i % 400 == 0)
68: 213              {
214                 cout<<endl;
215               cout<<i<<endl;
216               ++days_total;
217              }
69: 218           }
70: 219           }
71: 220       }
221
222     //////////////////////////Output//////////////////////////////////
223
72:
225        cout<<endl;
226        cout<<"\nThe total days in between your dates are:
    "<<days_total<<endl;
227     cout<<endl;
```

```
229         cin.get();
230         cin.ignore();
231         return 0;
```

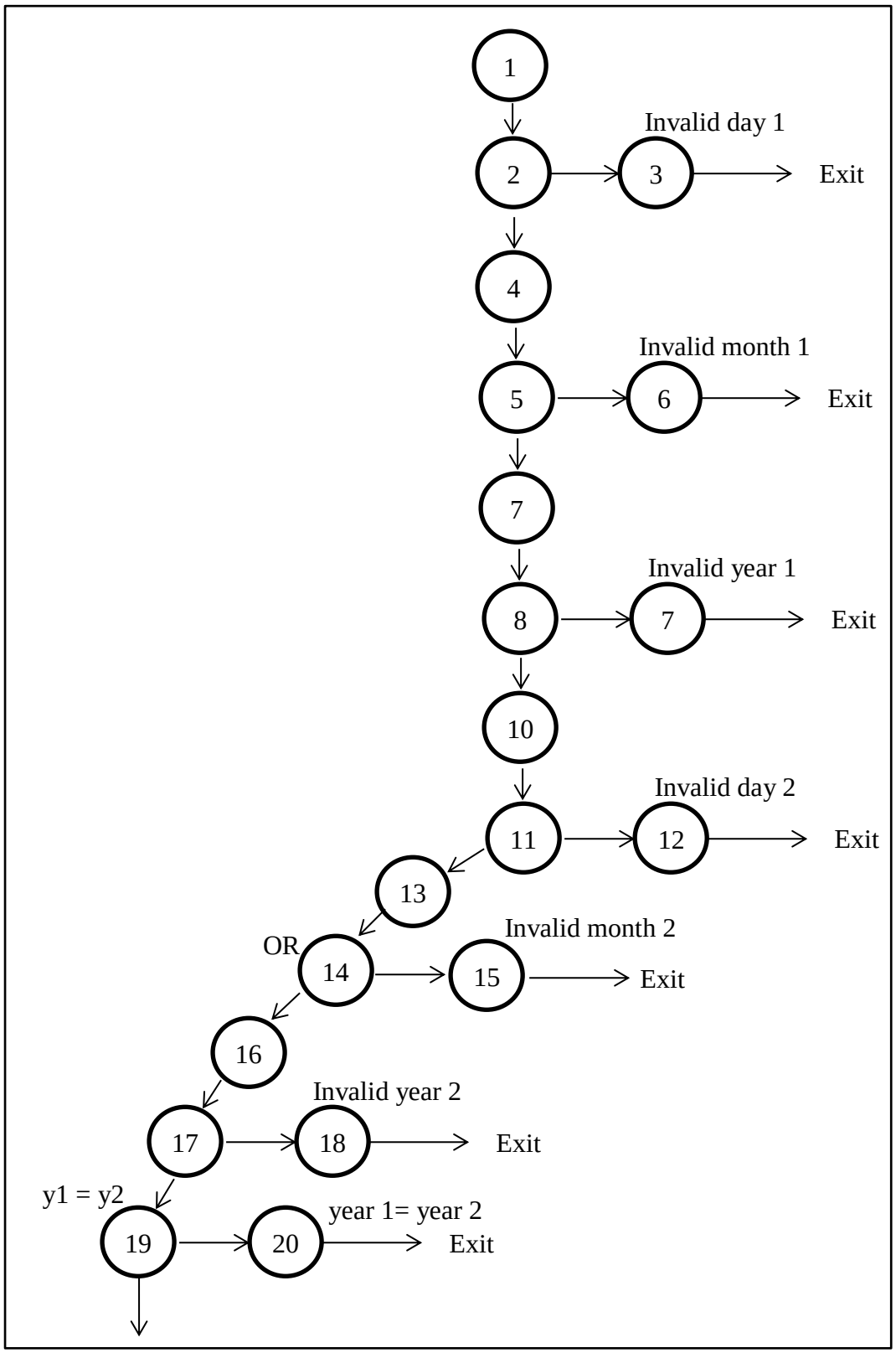

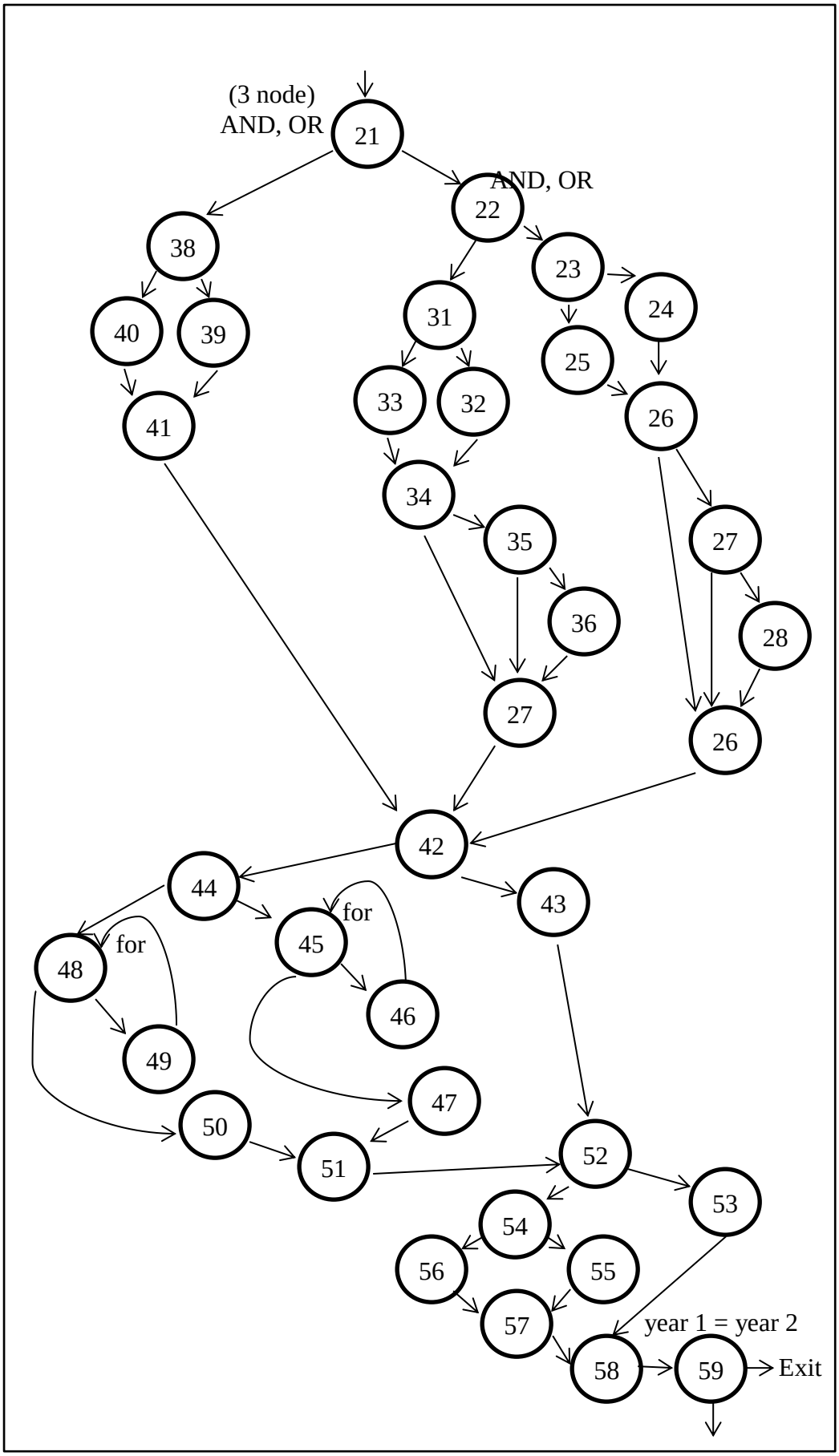
(3 node)
AND, OR
21
AND, OR
22
38
23
40
39
31
24
25
41
33
32
26
34
35
27
36
28
27
26
42
44
43
for
45
for
48
46
49
47
50
51
52
53
54
56
55
57
year 1 = year 2
58
59
Exit

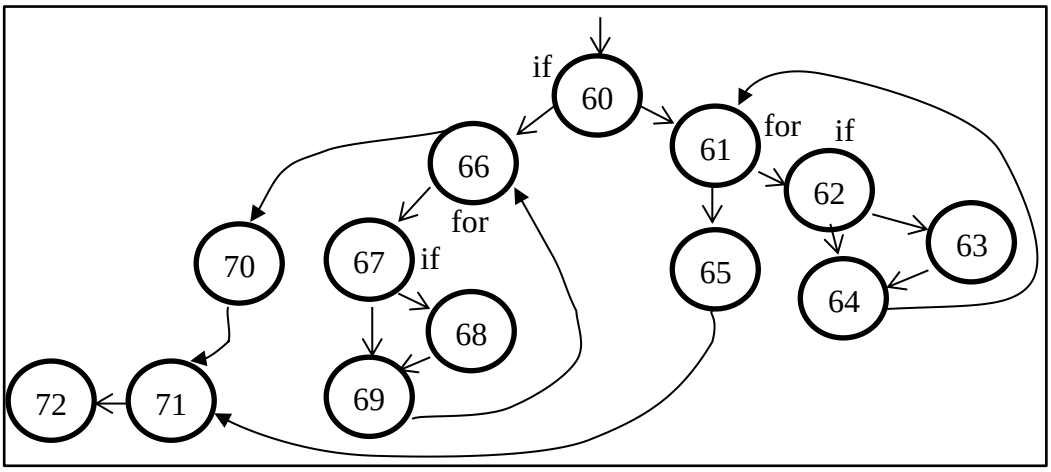

The CFG of the Calculating Number of Days between Two Dates (`NumDays`)

10. Calculates What Day of the Week for a Specific Date Source Code Program and CFG (`CalDay`)

1 month 12
1 day 31
1900 year 2058
The possible values of the output may be [Sunday, Monday, Tuesday, Wednesday, Thursday, Friday, Saturday, Invalid date].

```
/*Program to compute day of the week*/
/*Header Files*/
#include<stdio.h>
#include<conio.h>
1.  void main()
2.  {
3.  int day,month,year,century,Y,y1,M,date,validDate=0,leap=0;
4.  clrscr();
5.  printf("Enter day:");
6.  scanf("%d",&day);
7.  printf("Enter month:");
8.  scanf("%d",&month);
9.  printf("Enter year (between 1900 and 2058):");
10. scanf("%d",&year);
11. /*Check whether the date is valid or not*/
12. if(year>=1900&&year<=2058) {
13. if(year%4==0) { /*Check for leap year*/
14. leap=1;
15.     if((year%100)==0&&(year%400)!=0) {
16. leap=0;
17. }
18. }
19. if(month==4||month==6||month==9||month==11){
20. if(day>=1&&day<=30) {
```

```
validDate=1;
}
else {
validDate=0;
}
}
else if(month==2){
if(leap==1&&(day>=1&&day<=29)) {
validDate=1;
}
else if(day>=1&&day<=28) {
validDate=1;
}
else {
validDate=0;
}
}
else if((month>=1&&month<=12)&&(day>=1&&day<=31)){
validDate=1;
}
else {
validDate=0;
}
}
if(validDate) { /*Calculation of Day in the week*/
if(year>=1900&&year<2000){
century=0;
y1=year-1900;
}
26 else {
century=6;
y1=year-2000;
}
27. Y=y1+(y1/4);
28.  if(month==1) {
29. if(leap==0) {
30. M=0; /*for non-leap year*/
}
31. else {
M=6; /*for leap year*/
}
32. }
33. else if(month==2){
34. if(leap==0) {
35. M=3; /*for non-leap year*/
}
36. else {
M=2; //for leap year
}
37. }
```

```
38. else if((month==3)||(month==11)) {
39. M=3;
}
40. else if((month==4)||(month==7)) {
41. M=6;
}
42. else if(month==5) {
43. M=1;
}
44. else if(month==6) {
45. M=4;
}
46. else if(month==8) {
47. M=2;
}
48. else if((month==9)||(month==12)) {
49. M=5;
}
50. else {
M=0;
}
51. date=(century+Y+M+day)%7;
52. if(date==0) { /*Determine the day of the week*/
53. printf("Day of the week for [%d:%d:%d] is Sunday",day,month,year);
}
54. else if(date==1) {
55. printf("Day of the week for [%d:%d:%d] is Monday",day,month,year);
. }
56. else if(date==2) {
        57. printf("Day of the week for [%d:%d:%d] is
    Tuesday",day,month,year);
        }
        58. else if(date==3) {
        59. printf("Day of the week for [%d:%d:%d] is
    Wednesday",day,month,year);
        . }
        60. else if(date==4) {
        61. printf("Day of the week for [%d:%d:%d] is
    Thursday",day,month,year);
        . }
        62. else if(date==5) {
        63. printf("Day of the week for [%d:%d:%d] is
    Friday",day,month,year);
        . }
        64. else {
        printf("Day of the week for [%d:%d:%d] is
    Saturday",day,month,year);
         }
         }
        65. else {
```

```
116.          printf("The date entered [%d:%d:%d] is invalid",day,month,year);
117.          }
118.         66. getch();
119.          }
```

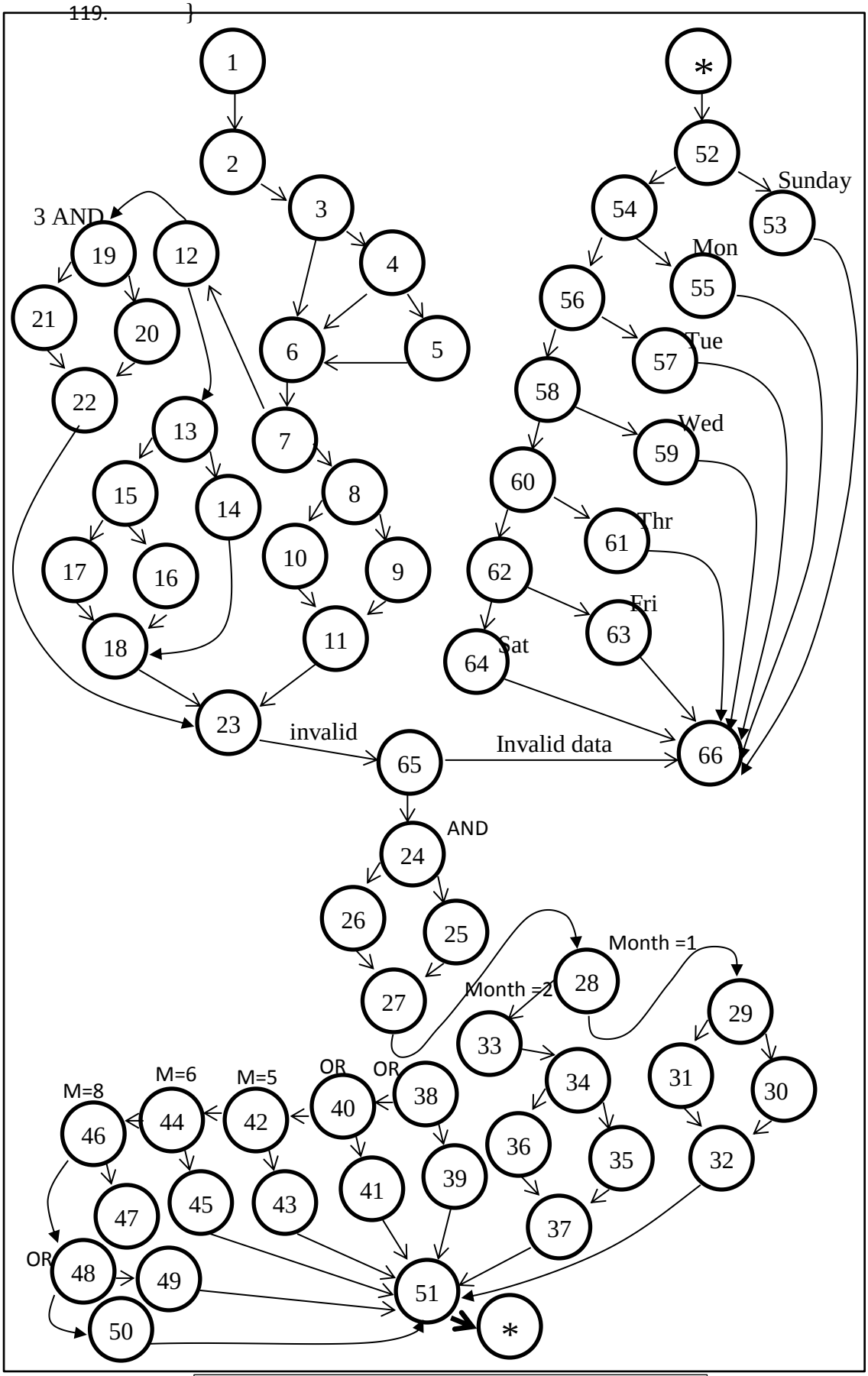

The CFG of the `CalDay` Program

11. The AllTrue program `(AllTrue32)`:

```
1.  1: AllTrue(boolean[] a)
2.      {
3.      boolean alltrue = true;
4.     2: for (i = 0; i < 64; i++)
5.       {
6.     3:  alltrue = alltrue && a[i];
7.     4: }
8.     5: if (alltrue)
9.     6: {
10.       // target
11.    7: }
12. 8: }
```

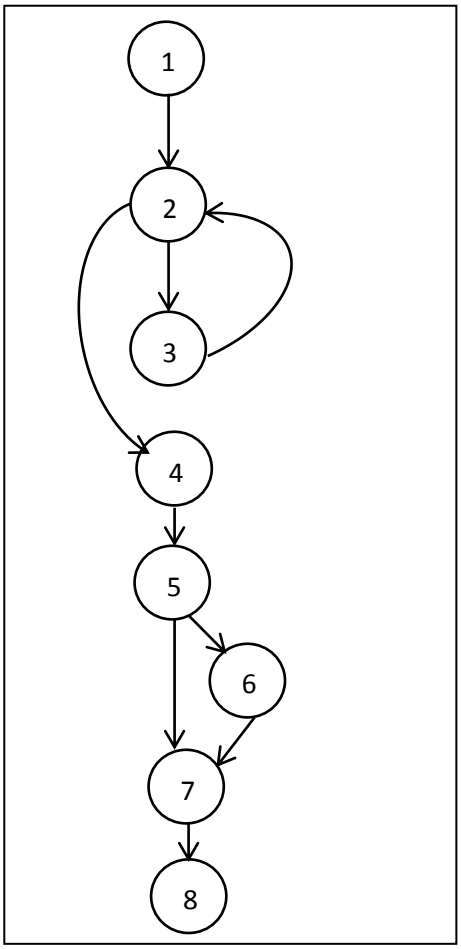

Supplement: S1 Dataset — (PDF) [file pone.0242812.s001.pdf]
